# Shugoshin forms a specialized chromatin domain at subtelomeres that regulates transcription and replication timing

**DOI:** 10.1038/ncomms10393

**Published:** 2016-01-25

**Authors:** Sanki Tashiro, Tetsuya Handa, Atsushi Matsuda, Takuto Ban, Toru Takigawa, Kazumi Miyasato, Kojiro Ishii, Kazuto Kugou, Kunihiro Ohta, Yasushi Hiraoka, Hisao Masukata, Junko Kanoh

**Affiliations:** 1 Institute for Protein Research, Osaka University, 3-2 Yamadaoka, Suita, Osaka 565-0871, Japan; 2 Department of Biological Sciences, Graduate School of Science, Osaka University, Toyonaka, Osaka 560-0043, Japan; 3 Advanced ICT Research Institute Kobe, National Institute of Information and Communications Technology, Kobe, Hyogo 651-2492, Japan; 4 Graduate School of Frontier Biosciences, Osaka University, Suita, Osaka 565-0871, Japan; 5 Department of Life Sciences, The University of Tokyo, Meguro-ku, Tokyo 153-8902, Japan

## Abstract

A chromosome is composed of structurally and functionally distinct domains. However, the molecular mechanisms underlying the formation of chromatin structure and the function of subtelomeres, the telomere-adjacent regions, remain obscure. Here we report the roles of the conserved centromeric protein Shugoshin 2 (Sgo2) in defining chromatin structure and functions of the subtelomeres in the fission yeast *Schizosaccharomyces pombe*. We show that Sgo2 localizes at the subtelomeres preferentially during G_2_ phase and is essential for the formation of a highly condensed subtelomeric chromatin body ‘knob'. Furthermore, the absence of Sgo2 leads to the derepression of the subtelomeric genes and premature DNA replication at the subtelomeric late origins. Thus, the subtelomeric specialized chromatin domain organized by Sgo2 represses both transcription and replication to ensure proper gene expression and replication timing.

## Abstract (web summary)

A chromosome is composed of structurally and functionally distinct domains. Here, Tashiro *et al*. report that the conserved centromeric protein Sgo2 localizes at the subtelomeres preferentially during G_2_ phase and is essential for the formation of a highly condensed subtelomeric chromatin body “knob”.

## Graphical abstract

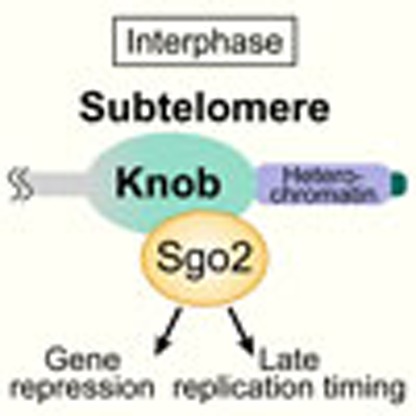

Eukaryotic chromosomes consist of various chromatin domains that contribute to genomic integrity and functions. The telomere is a specialized chromatin domain located at chromosome ends that contains repetitive nucleotide sequences called ‘telomere repeats' and associated proteins. The telomere protects the chromosome ends and ensures proper chromosome movement during both mitosis and meiosis for genome stability^1^,^2^,^3^. The subtelomere is the chromosomal region located adjacent to the telomere. Subtelomeric (ST) DNA contains multiple elements that share high sequence similarity among the subtelomeres of an individual organism.

In the budding yeast *Saccharomyces cerevisiae*, the subtelomeres are highly variable in length and commonly contain an X element and tandem copies of a Y' element, which includes the open reading frame (ORF) of an RNA helicase gene^4^. In humans, the subtelomeres comprise a complex mosaic of >40 duplicated segments with a variety of ORFs^5^. Although the sequencing is not yet complete ( http://www.pombase.org/status/sequencing-status), the subtelomeres of *S. pombe* also contain a mosaic region of multiple segments of the highly similar sequences that spans ∼50 kb of the telomere-proximal site^6^,^7^—a site containing multiple ORFs of common genes including the RecQ-type DNA helicase genes (*tlh1*^+^and *tlh2*^+^). In addition, the chromatin structure of the *S. pombe* subtelomere is composed of two distinct parts: heterochromatin and ST chromatin. The heterochromatin contains histone H3 with methylated Lys9 (H3K9me) and HP1 homologues, and spans ∼45–80 kb of the telomere-proximal site^8^,^9^,^10^. The adjacent telomere-distal ST chromatin spans ∼50 kb and shows low sequence similarity among the subtelomeres, but shares a common feature of low levels of methylated and acetylated histones^11^. In contrast to the substantial accumulation of knowledge on telomeres, not much attention has been paid to subtelomeres, and their regulation and physiological function remain largely unknown.

The centromere is a crucial domain for the faithful segregation of chromosomes during cell division. Shugoshin (Sgo) is a conserved centromeric protein that protects centromeric cohesion and promotes loading of the chromosomal passenger complex (CPC) onto centromeres^12^,^13^,^14^,^15^,^16^,^17^,^18^,^19^,^20^. *S. pombe* possesses two Sgo paralogues: Sgo1 and Sgo2. Sgo1 is expressed specifically during meiosis I, recruits PP2A phosphatase to the centromeres and thereby counteracts the casein kinase 1-dependent cleavage of cohesin^13^,^21^; in contrast, Sgo2 is ubiquitously expressed during the mitotic and meiotic cell cycle, and plays an important role in recruitment of CPC to the centromeres for proper chromosome segregation in M phase^18^. Intriguingly, during interphase, the accumulation of Sgo2 at the centromeres decreases and, instead, a substantial fraction appears at the vicinity of the telomeres^17^,^18^,^22^. However, details of the regulation and localization of Sgo2 during interphase, in addition to its physiological roles at the telomere vicinity, remain largely unknown.

In this study, we reveal new roles of Sgo2 at the subtelomeres in *S. pombe*. We show that Sgo2 associates with the subtelomeres preferentially during G_2_ phase and is essential for the formation of a highly condensed interphase-specific chromatin structure at the subtelomeres. Furthermore, Sgo2 regulates ST gene expression by reducing the occupancy of RNA polymerase II (RNAPII) and maintains normal timing of DNA replication by limiting loading of a replication initiation factor to the late origins within the subtelomeres. Thus, Sgo2 defines the chromatin structure and functions of subtelomeres during interphase in *S. pombe*.

## Results

### Sgo2 associates with the subtelomeres of chromosomes 1 and 2

**Figure 1:**
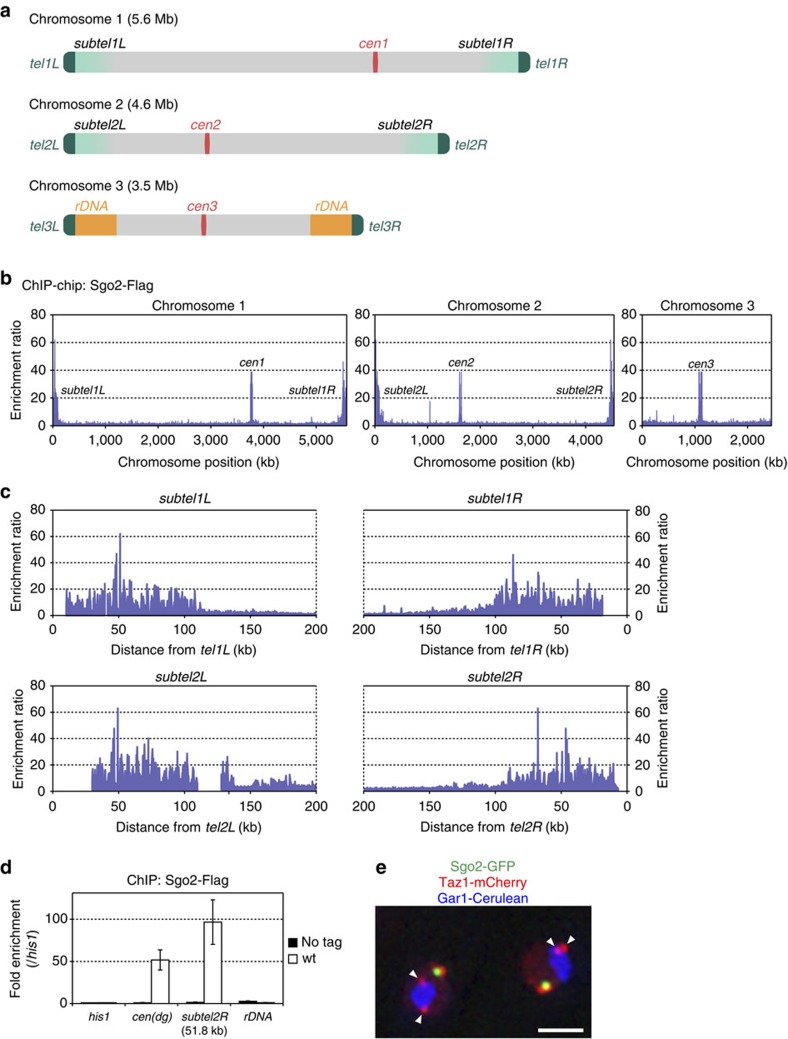
Sgo2 associates with the whole subtelomeres of chromosomes 1 and 2. (**a**) Schematic illustration of *S. pombe* chromosomes. Chromosomes 1 and 2 contain telomere-proximal common sequences that are highly similar among the subtelomeres, whereas chromosome 3 contains rDNA repeats close to the telomeres. (**b**) ChIP–chip analysis of the genome-wide distribution of Sgo2-Flag in logarithmically growing wt cells. It is noteworthy that the probes used in the tiling array did not encompass regions outside the rDNA repeats on chromosome 3. (**c**) Magnification of the ChIP–chip data for the subtelomeric regions shown in **b**. Gaps in the data represent regions not included in the chips due to a lack of sequence information. (**d**) ChIP analyses of Sgo2-Flag localization at the rDNA repeats in asynchronous cultures (predominantly in interphase). Relative fold enrichment, normalized to the signal at the *his1*^+^ locus, is shown. No tag indicates the negative control for the ChIP analyses; *cen(dg)* indicates the *dg* repeats at the centromeres; *subtel2R-51.8K* indicates 51.8 kb from *tel2R*. Error bars indicate the s.d. (*n*=3). (**e**) Subnuclear localization of Sgo2-GFP. Sgo2-GFP, Taz1-mCherry (telomere) and Gar1-Cerulean (nucleolus) signals are shown in green, red and blue, respectively. Arrowheads indicate the telomeres of chromosome 3. Scale bar, 2 μm.

To characterize the precise distribution of Sgo2 across the three chromosomes of *S. pombe* (Fig. 1a), we performed chromatin immunoprecipitation (ChIP)–chip analyses of Flag-tagged Sgo2 in logarithmically proliferating cells, which were primarily in interphase. Sgo2-Flag was enriched not only at the centromeres but also at the subtelomeres of both chromosomes 1 and 2 (*subtel1L*, *1R*, *2L* and *2R*), extending >100 kb from each telomere (Fig. 1b,c). These Sgo2-enriched regions encompassed the clusters of non-essential genes^23^ and partially overlapped with the telomere-adjacent ST heterochromatin, which contains H3K9me and the heterochromatin protein Swi6/HP1 (refs 8, 9).

It was not clear whether Sgo2 also localizes near the ends of chromosome 3, because the *S. pombe* genome database does not cover the regions external to the rDNA repeats that are located near both telomeres of chromosome 3 (*tel3*)^7^ (Fig. 1a). However, Sgo2 was not enriched at the rDNA repeats of chromosome 3 in the ChIP assay (Fig. 1d). Furthermore, Sgo2 was barely detectable by microscope in the close vicinity of *tel3*, as indicated by the telomere-binding protein Taz1 (ref. 24) that is associated with the nucleolus protein Gar1 (ref. 25 and Fig. 1e). Thus, it is unlikely to be that Sgo2 accumulates near *tel3*. The absence of Sgo2 near *tel3* is possibly due to the lack of the highly similar ST DNA sequences in chromosome 3 of the wild-type (wt) strain used in this study (Supplementary Fig. 1).

### Sgo2 associates with the subtelomeres preferentially in G_2_

**Figure 2:**
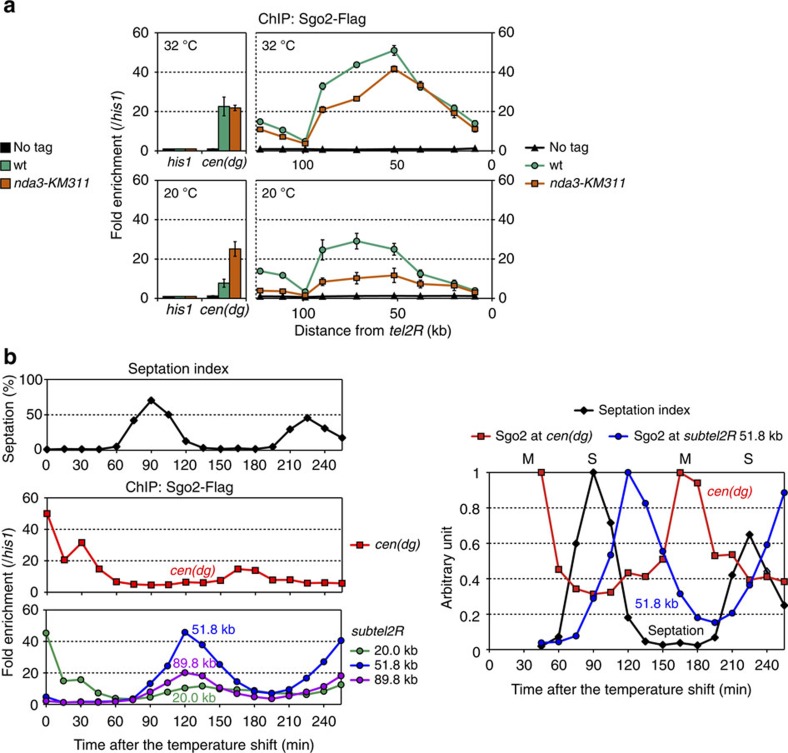
Sgo2 associates with the subtelomeres preferentially in G_2_ phase. (**a**) ChIP analyses of Sgo2-Flag localization at *subtel2R* in M phase. Cold-sensitive *nda3-KM311* mutant and wt (control) cells were grown at 32 °C (permissive temperature) or incubated at 20 °C for 12 h (restrictive temperature, M-phase-arrested) in YPD medium. Relative fold enrichment at *subtel2R* (right) and *cen* (*dg*) (left), normalized to the signal at the *his1*^+^ locus, is shown. It is noteworthy that the DNA sequence of *subtel2R* within ∼50 kb of the end of the chromosome is highly similar to that of the other subtelomeres^7^; therefore, the analysis cannot differentiate these common telomere-proximal subtelomeric regions. No tag indicates the negative control for ChIP. Error bars indicate the s.d. (*n*=3). (**b**) ChIP analyses of Sgo2-Flag localization at *subtel2R* during the cell cycle. Temperature-sensitive *cdc25-22* mutant cells were arrested at the G_2_/M boundary at 35.5 °C (restrictive temperature) and then released from arrest by shifting to 25 °C (permissive temperature). Cell cycle progression was monitored by the septation index (left, top). Relative fold enrichment at *cen* (*dg*) (left, middle) and at 20.0, 51.8 and 89.8 kb from the telomere repeats in *subtel2R* (left, bottom), normalized to that at the *his1*^+^ locus, is shown, together with a summary of the relative Sgo2 enrichment at *cen* (*dg*) and at the 51.8 kb locus (*subtel2R*) (right). The time points 0, 15 and 30 min are omitted from the graph because of a lack of periodicity as compared with later time points, possibly due to non-physiological cell cycle arrest caused by the *cdc25-22* mutation.

We next examined the cell cycle dependency of the Sgo2 association with the subtelomeres. Cells were arrested in M phase via the *nda3-KM311* mutation^26^ and the localization of Sgo2 was analysed by ChIP. Sgo2 association with *subtel2R* was slightly weaker in *nda3-KM311* cells even at the permissive temperature (32 °C), as compared with wt cells (Fig. 2a upper); this difference was enhanced when cells were incubated at the restrictive temperature (20 °C), which arrested the *nda3* mutant in M phase, while the wt strain remained asynchronously growing (Fig. 2a lower). In contrast, the centromeric localization of Sgo2 was considerably increased in the *nda3* mutant (Fig. 2a lower), which is consistent with previous findings that Sgo2 accumulates at centromeres during M phase^17^,^18^,^22^. Furthermore, time-lapse microscopy revealed that Sgo2 dissociated from the vicinity of the telomeres immediately before chromosome segregation was initiated (Supplementary Fig. 2, 2–4 min.). These data suggest that Sgo2 associates with the subtelomeres preferentially during interphase.

To pinpoint the cell cycle stage during interphase at which Sgo2 accumulates at the subtelomeres, cells were synchronized by means of the *cdc25-22* mutation^27^, and the localization of Sgo2 at the centromeres and at the three different loci (20.0, 51.8 and 89.8 kb from *tel2R*) in *subtel2R* were analysed by ChIP. Enrichment of Sgo2 at the subtelomeres reached its maximum peak in the middle of G_2_ phase, which is between the peak of the septation index (S phase) and the peak of centromeric localization of Sgo2 (M phase) (Fig. 2b). These results indicate that the localization of Sgo2 is dynamically regulated during the cell cycle, and that Sgo2 associates with the subtelomeres preferentially in the middle of G_2_ phase.

### H2A phosphorylation is important for the Sgo2 localization

**Figure 3:**
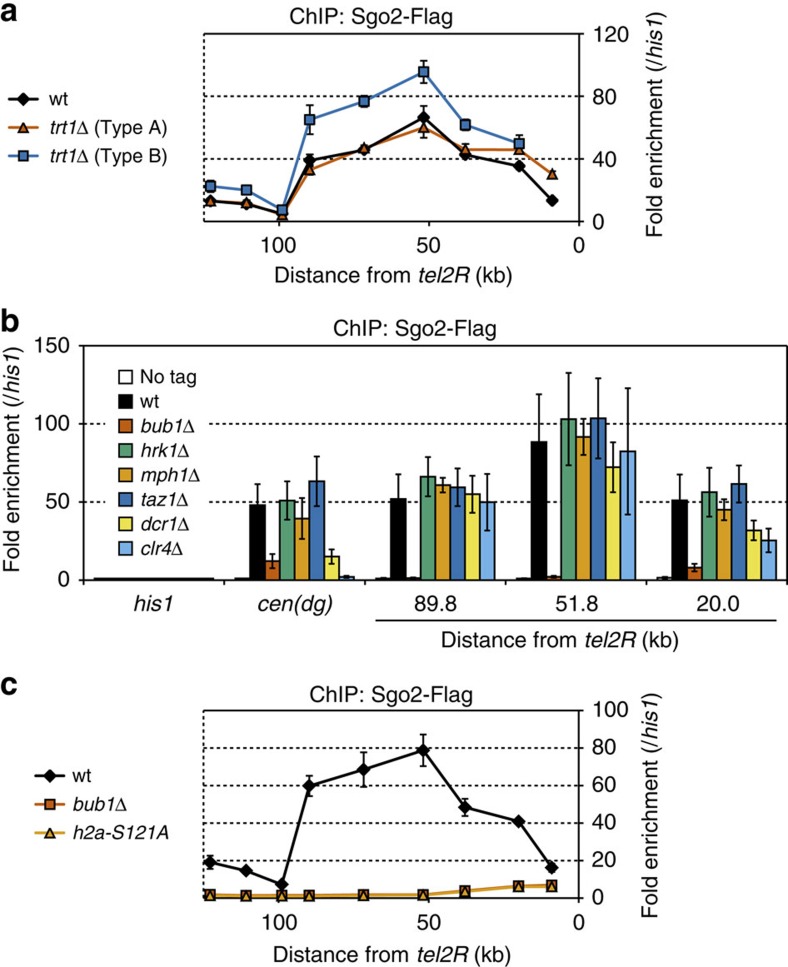
H2A-S121 phosphorylation by Bub1 is important for Sgo2 association with the subtelomeres. (**a**) ChIP analyses of Sgo2-Flag localization at *subtel2R* in *trt1*Δ survivor cells (Types A and B). Relative fold enrichment at *subtel2R*, normalized to the signal at the *his1*^+^ locus, is shown. It is noteworthy that the most telomere-proximal data point, which corresponds to the region lost in Type B, is missing for Type B. (**b**) ChIP analyses of Sgo2-Flag localization in various deletion mutants. Relative fold enrichment at *subtel2R* and the centromeres (*dg*), normalized to the signal at the *his1*^+^ locus, is shown. (**c**) ChIP analysis of Sgo2-Flag localization at *subtel2R* in the *bub1*Δ and *h2a-S121A* mutants. The relative fold enrichment at *subtel2R* as normalized by the signals at the *his1*^+^ locus is plotted. The error bars indicate the s.d. (*n*=3).

Next, we investigated the requirements for localization of Sgo2 at the subtelomeres. First, we examined whether the telomeric structure is required for Sgo2 to associate with the subtelomeres. Deletion of the telomerase catalytic subunit Trt1 generally leads to cell death due to the loss of telomeric repeat DNA and de-protection of the chromosome ends^28^. However, a fraction of *trt1*Δ cells survive if all of the chromosomes become self-circularized via chromosomal end fusion at the subtelomeres^29^,^30^. A survivor that has lost ∼9 kb of subtelomeres from the chromosome ends is called Type A, whereas one that has lost ∼15 kb is called Type B^31^. We found that Sgo2 remained attached to the subtelomeres in both types of *trt1*Δ survivor lacking telomeric DNA (Fig. 3a), indicating that the telomeric structure is dispensable for the association of Sgo2 with the subtelomeres.

We also examined whether well-established chromatin-related factors are required for localization of Sgo2 at the subtelomeres, including centromeric proteins that play a role in activation of the spindle assembly checkpoint such as Bub1 (ref. 22), Hrk1 (ref. 32) and Mph1 (ref. 33); the core telomere-binding protein Taz1 (ref. 24); and the heterochromatin factors Clr4 and Dcr1 (refs 8, 9). Among the respective deletion mutants, only the *bub1*^+^ deletion abolished the ST localization of Sgo2 almost completely (Fig. 3b and Supplementary Fig. 3). Bub1 is the sole protein kinase that phosphorylates serine 121 of histone H2A (H2A-S121) (Supplementary Fig. 4) and this histone modification is important for the association of Sgo2 with chromatin^22^. Substituting serine 121 of H2A with a non-phosphorylatable alanine (*h2a-S121A*) resulted in almost the same level of removal of Sgo2 from the subtelomeres as observed in *bub1*Δ (Fig. 3c), although the amount of Sgo2 protein in *bub1*Δ, *h2a-S121A* and wt cells was comparable (Supplementary Fig. 5). Moreover, localization of Sgo2 at the subtelomeres was greatly decreased in cells carrying the *bub1-KD* (kinase-dead) mutation to almost the same level as observed in the *h2a-S121A* mutant (Supplementary Fig. 6). These data indicate that the phosphorylation of H2A-S121 by Bub1 is important for the association of Sgo2 with subtelomeres, whereas the telomeric structure, the activation of spindle assembly checkpoint and the heterochromatin structure are dispensable.

### Sgo2 is crucial for the formation of highly condensed knobs

The primary function of Sgo2 is to recruit CPC to centromeres for the bipolar attachment of kinetochores to spindle microtubules during metaphase in *S. pombe*^17^,^18^. However, our ChIP analyses revealed that any of the CPC components (Ark1, Bir1, Pic1 and Nbl1) is hardly localized at the subtelomeres throughout the cell cycle, in stark contrast to their apparent accumulation at the centromeres during M phase (Supplementary Fig. 7). We therefore explored the idea that Sgo2 has a new function at the subtelomeres in interphase.

**Figure 4:**
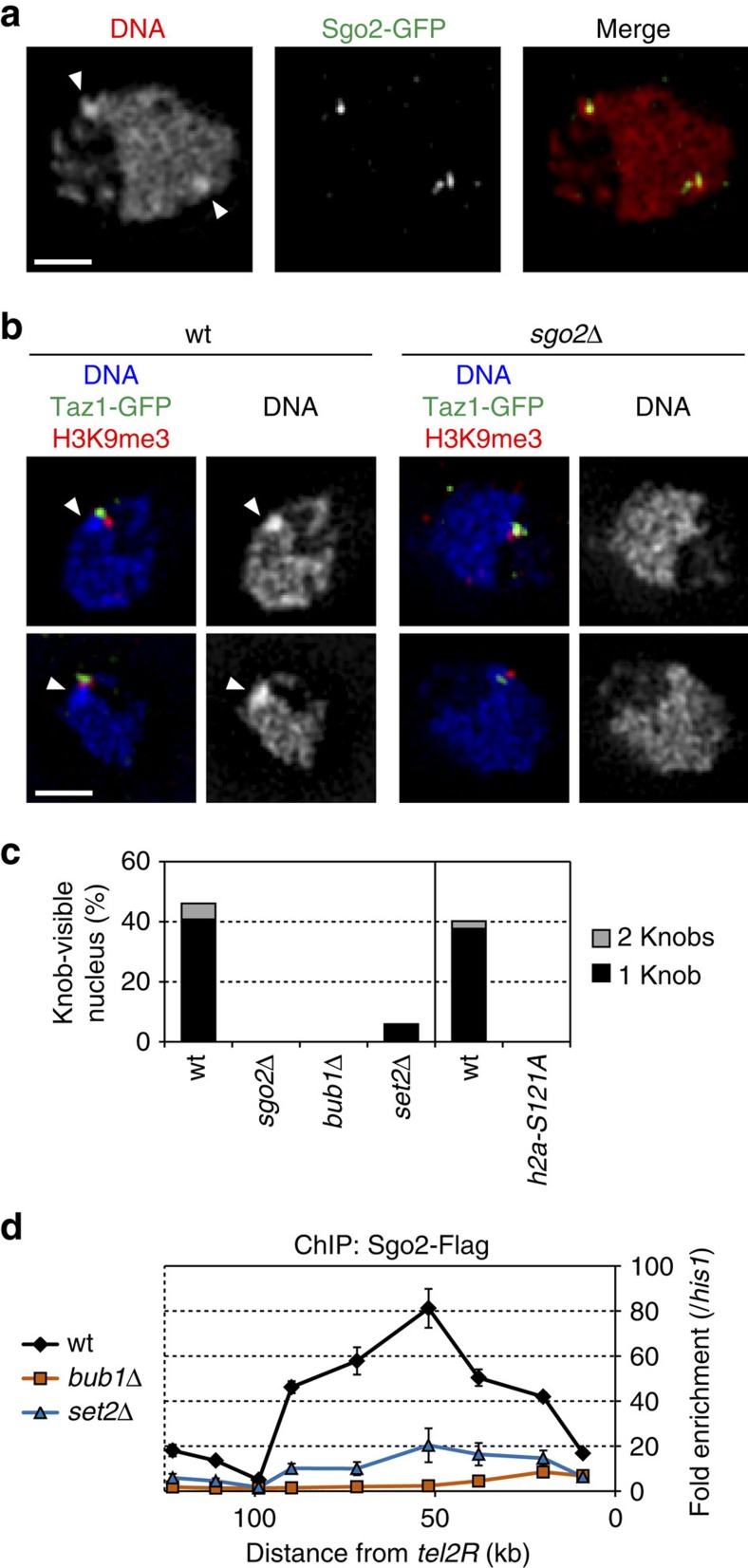
Sgo2 is required for the formation of knobs. (**a**) Co-localization of Sgo2-GFP with knobs during interphase. Cells were grown in EMM at 26 °C and then fixed in formaldehyde (see Methods). 3DSIM images of single optical sections for DAPI and Sgo2-GFP (from immunostaining using anti-GFP antibody) signals in an interphase cell are shown. Arrowheads indicate knobs with intense DAPI signals. Scale bar, 1 μm. (**b**) Knobs are localized near Taz1-GFP (telomere) and H3K9me3 (subtelomeric heterochromatin) in wt cells, but are absent in *sgo2*Δ cells. 3DSIM images of single optical sections were taken as in **a**. Arrowheads indicate knobs. Scale bar, 1 μm. (**c**) Sgo2 association with subtelomeres is required for knob formation. Frequencies of knob formation in each strain during exponential growth are shown. (**d**) Sgo2 association with subtelomeres is substantially decreased in the absence of Set2. Sgo2-Flag localization at *subtel2R* in *bub1*Δ and *set2*Δ cells was analysed by ChIP. Relative fold enrichment at *subtel2R*, normalized to the signal at the *his1*^+^ locus, is shown. Error bars indicate the s.d. (*n*=3).

‘Knobs' are highly condensed chromatin bodies in *S. pombe* that are intensely stained with the DNA-labelling dye 4′,6-diamidino-2-phenylindole (DAPI). They are located in the vicinity of the telomeres, more precisely adjacent to the ST heterochromatin (as indicated by the H3K9me3 signal adjacent to that of Taz1), specifically during interphase^34^ (see Fig. 4b wt). Here we found that the subnuclear localization of Sgo2–green fluorescent protein (GFP) always overlapped with the knobs (Fig. 4a), and that the knobs were completely lost in *sgo2*Δ cells (Fig. 4b,c). Consistent with this, *bub1*Δ and *h2a-S121A* mutant cells, in which Sgo2 was dissociated from the subtelomeres (Fig. 3c), also completely lacked knobs (Fig. 4c). These data demonstrate that the association of Sgo2 with subtelomeres plays a crucial role in the knob formation.

It has been shown that the frequency of the knob formation is substantially decreased by the deletion of Set2 (ref. 34), which is the methyltransferase for H3K36 (refs 35, 36 and Fig. 4c). ChIP analyses revealed that localization of Sgo2 at the subtelomeres also decreased markedly in the *set2*Δ mutant as compared with the wt strain (Fig. 4d). These data further support our conclusion that the knob formation is dependent on localization of Sgo2 at the subtelomeres.

### Sgo2 represses transcription of the ST genes

**Figure 5:**
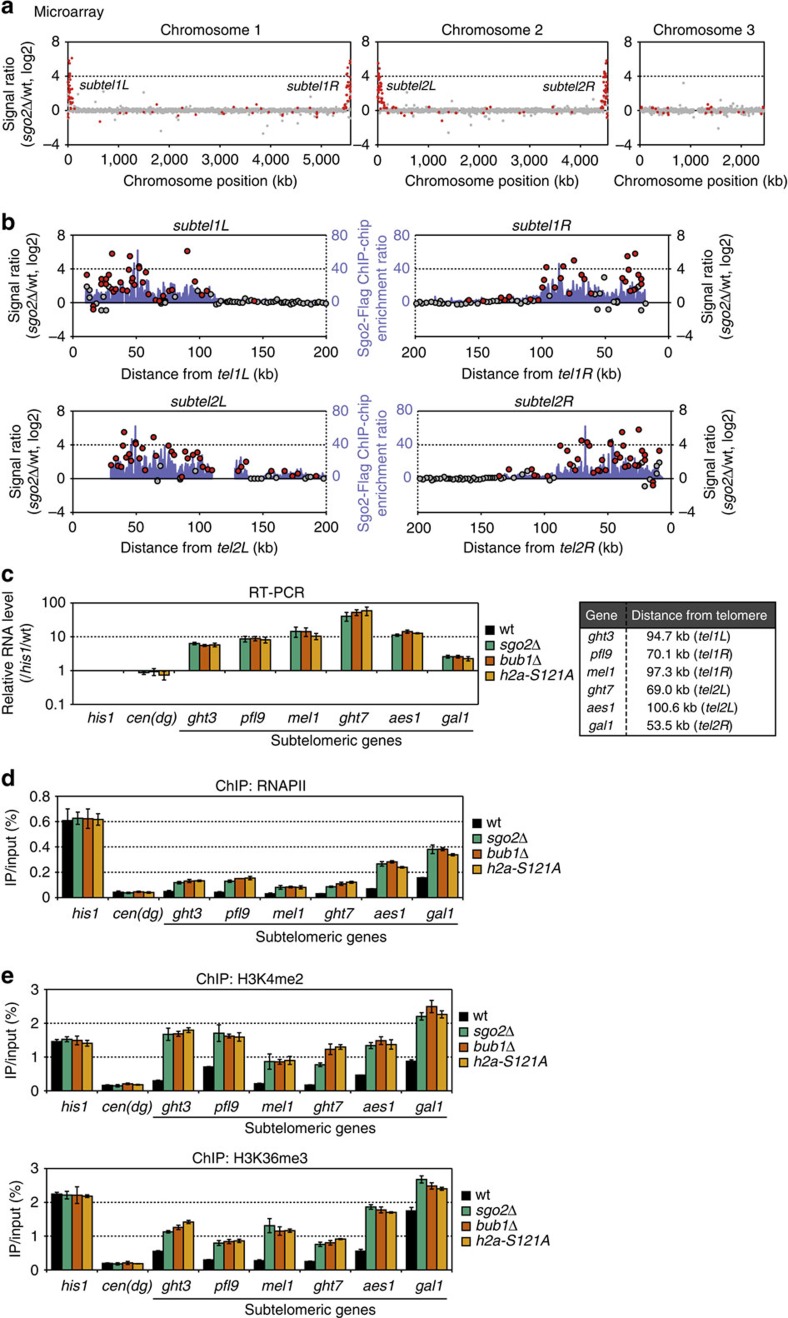
Sgo2 represses transcription of the subtelomeric genes. (**a**) Microarray analysis of genome-wide changes in RNA expression caused by Sgo2 deletion. The signal ratio of each probe on the array is shown. Red dots and grey dots indicate significant (*P*<0.002 for an increase and *P*>0.998 for a decrease) and insignificant changes, respectively. The *P*-value was calculated as change *P*-value (two-sided) by Affymetrix GCOS1.4 with the default settings. (**b**) Magnified views of the subtelomeric regions in **a**. Localization of Sgo2 (from the ChIP–chip analyses) is indicated by the pale blue colour. (**c**) Association of Sgo2 with the subtelomeres is required for subtelomeric gene repression. The transcript levels of the subtelomeric genes and the centromeric repeats (*dg*) in wt, *sgo2*Δ, *bub1*Δ and *h2a-S121A* cells were analysed by quantitative reverse transcriptase–PCR (RT–PCR). The locations of the genes are shown in the box. Each value was normalized first to that of *his1*^+^ and then to the wt value. (**d**) Occupancy of RNAPII at the subtelomeric gene loci is increased in *sgo2*Δ, *bub1*Δ and *h2a-S121A* cells. ChIP analyses of RNAPII at the subtelomeric gene loci and at the centromeric repeats (*dg*) are shown. The recovery of immunoprecipitated DNA relative to total input DNA was determined by quantitative PCR. (**e**) ChIP analyses of the levels of H3K4me2 (upper) and H3K36me3 (lower). The recovery of immunoprecipitated DNA relative to total input DNA was measured by quantitative PCR. Error bars indicate the s.d. (*n*=3).

As highly condensed chromatin is generally expected to be inhibitory to transcription^37^, we investigated the effects of the Sgo2-dependent knob formation on gene expression. Genome-wide expression profiles were obtained by microarray analyses using poly(A)-containing RNAs that were purified from asynchronously growing wt and *sgo2*Δ cells. We found that the transcript levels of genes at *subtel1L*, *1R*, *2L* and *2R* were higher in *sgo2*Δ cells than in wt cells, whereas gene expression was not strongly affected, if at all, by *sgo2*Δ in other chromosomal regions (Fig. 5a). Notably, the locations of the genes affected by *sgo2*Δ correlated with the ST distribution of Sgo2, although the expression of some ST genes were barely affected (Fig. 5b and Supplementary Fig. 8), which may be due to the unequal ST localization of Sgo2 and/or the difference in the promoter activity of each gene. These data imply that the localization of Sgo2 is important for the observed repressive effect on ST gene expression.

Indeed, reverse transcriptase-PCR (RT-PCR) analyses showed that the transcription of several ST genes was also de-repressed in the *bub1*Δ, *h2a-S121A* and *set2*Δ mutants, as observed in the *sgo2*Δ mutant, whereas the expression level of the centromeric repeats (*dg*) barely changed in these mutants (Fig. 5c and Supplementary Fig. 9a). Furthermore, ChIP analyses of RNAPII showed that RNAPII occupancy at these ST gene loci was considerably higher in the *sgo2*Δ, *bub1*Δ, *h2a-S121A* and *set2*Δ mutants than in the wt strain, whereas RNAPII occupancy at the centromeric *dg* was barely altered (Fig. 5d and Supplementary Fig. 9b). These data indicate that Sgo2 mediates gene repression at the transcriptional level specifically at the subtelomeres. Consistently, the levels of histone modifications associated with transcriptionally active chromatin, such as dimethylation of lysine 4 and trimethylation of lysine 36 in histone H3 (H3K4me2 and H3K36me3, respectively)^35^,^36^,^38^, were elevated at the ST gene loci in *sgo2*Δ, *bub1*Δ, and *h2a-S121A* cells as compared with wt cells (Fig. 5e and see Discussion).

### Sgo2 forms a new type of repressive chromatin domain

**Figure 6:**
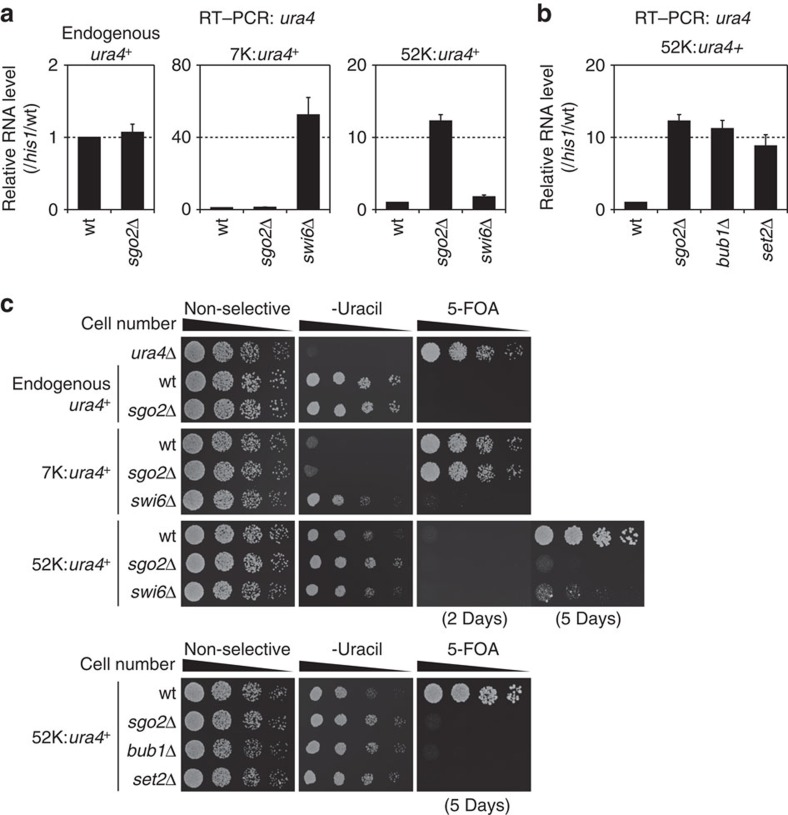
Sgo2 forms a transcriptionally repressed chromatin domain distinct from Swi6/HP1 heterochromatin. (**a**) The absence of Sgo2 and Swi6 has different effects on the repression of subtelomeric genes. The RNA level of the *ura4*^+^ gene in each strain was analysed by quantitative reverse transcriptase–PCR (RT–PCR); left, endogenous *ura4*^+^; middle, *ura4*^+^ inserted at 7 kb from *tel2R*; right, *ura4*^+^ inserted at 52 kb from *tel2R*. Each value of *ura4*^+^ relative to that of *his1*^+^ was re-normalized to the wt value. Error bars indicate the s.d. (*n*=3). (**b**) RNA expression of the *ura4*^+^ gene in *bub1*Δ and *set2*Δ cells was analysed as in **a**. (**c**) Growth assays of the strains used in **a** and **b**. Dilution series of cells were spotted on YES (non-selective complete medium), SD-uracil (lacking uracil) and 5-FOA (YES with 1 mg ml^−1^ 5-fluoroorotic acid, the counter-selection drug for *ura4*^+^) plates at 32 °C for 2 days unless otherwise indicated. The *ura4*Δ strain (top) was the control for the assay. It is noteworthy that 5 days incubation was necessary to see growth on 5-FOA for 52 K:*ura4*^+^ due to the lower level of gene repression as compared with the that 7 K:*ura4*^+^.

Swi6/HP1 is known to associate with chromatin in an H3K9me-dependent manner to form transcriptional repressive heterochromatin at the telomere-proximal region of each subtelomere^8^,^9^. Therefore, we compared the effects of *sgo2*^+^ and *swi6*^+^ deletion on gene repression by monitoring the expression of an exogenous gene inserted at the subtelomere. As compared with the wt strain, expression of the *ura4*^*+*^ gene placed 7 kb from the telomere (7 K:*ura4*^*+*^) increased remarkably in the *swi6*Δ mutant but not in the *sgo2*Δ mutant, indicating that the Swi6 heterochromatin is dominant to repress gene expression at the telomere-proximal region (Fig. 6a middle). In contrast, there was a remarkable increase in the expression of the *ura4*^*+*^ gene when it was placed 52 kb from the telomere (52 K:*ura4*^*+*^) in *sgo2*Δ cells, whereas only a small increase was observed in *swi6*Δ cells (Fig. 6a right). *bub1*Δ and *set2*Δ cells showed derepression of 52 K:*ura4*^*+*^ expression to levels similar to those in *sgo2*Δ cells (Fig. 6b), indicating that the ST localization of Sgo2 is required for this repressive effect. In contrast, expression of the endogenous *ura4*^*+*^ gene was unaffected in *sgo2*Δ cells (Fig. 6a, left), demonstrating that the Sgo2-mediated gene repression was region specific rather than gene specific. These observations were verified by monitoring cell growth on *ura4*^*+*^-selective media (Fig. 6c). Collectively, these results suggest that the association of Sgo2 with subtelomeres forms a new type of repressive chromatin domain that is distinct from the H3K9me-Swi6-mediated heterochromatin.

### Sgo2 regulates replication timing at the subtelomeres

*S. pombe* subtelomeres contain many DNA-replication origins that do not fire during early S phase^39^. The replication timing of some ST late origins are regulated by the telomere-binding proteins Taz1 and Rif1 (refs 40, 41); however, the overall regulatory mechanism underlying the ST replication timing remains elusive. Intriguingly, we noted that the clusters of late origins at the subtelomeres largely overlapped with the Sgo2-associated regions (Fig. 1c and Supplementary Fig. 10), which prompted us to examine whether Sgo2 might be involved in the control of replication timing at subtelomeres.

**Figure 7:**
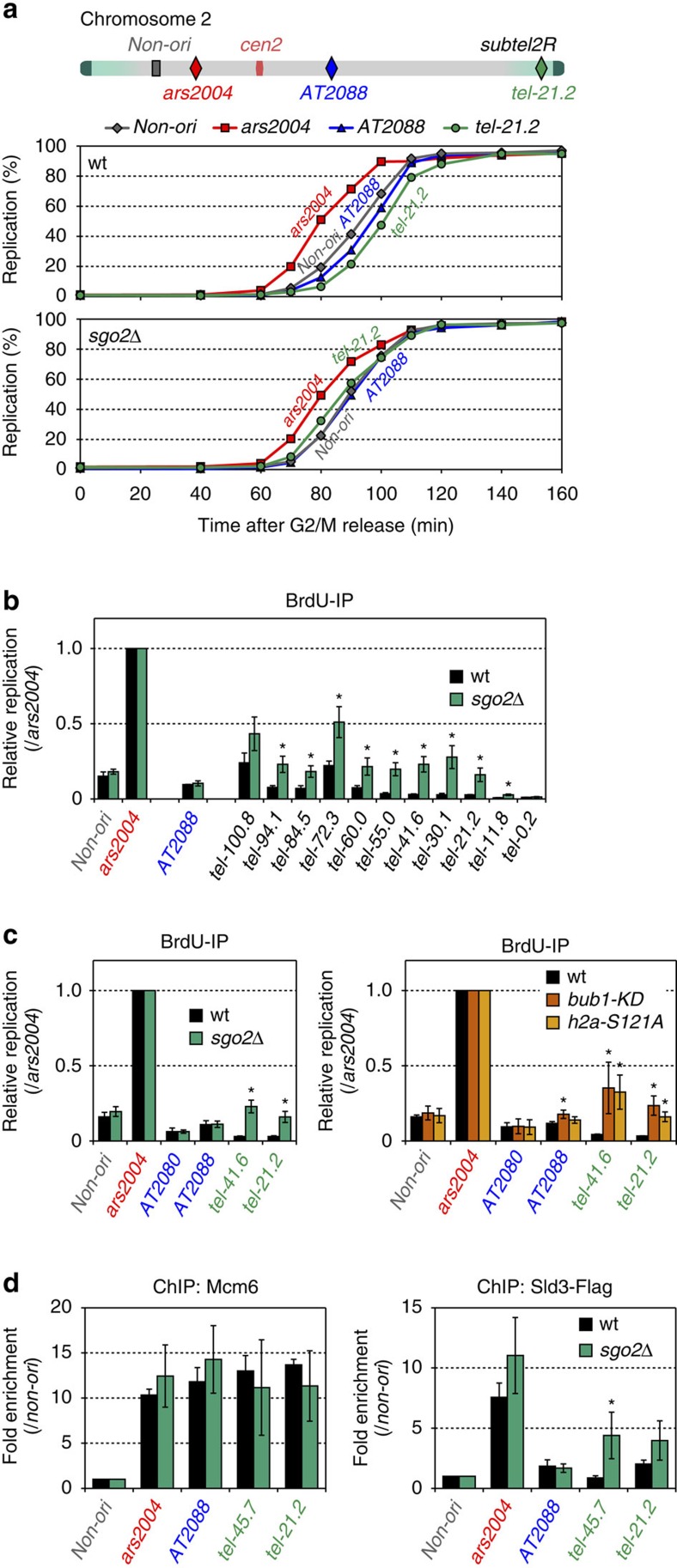
Sgo2 regulates replication timing at the subtelomeres by limiting Sld3 loading. (**a**) Replication kinetics of the early and late origins in wt and *sgo2*Δ cells. The locations of the origins on chromosome 2 are shown schematically (top). *ars2004* is an early origin and *AT2088* is a late origin. *tel-21.2* is located at 21.2 kb from *tel2R.* The non-origin region (*non-ori*) is located at 30 kb from *ars2004*. Temperature-sensitive *cdc25-22* mutant cells expressing thymidine kinase and the nucleotide transporter were arrested at the G_2_/M boundary at the restrictive temperature (36 °C) and then released from arrest by a temperature shift to 25 °C in the presence of BrdU (200 μM). At the indicated time points, the amount of HL DNA and LL DNA, which were separated by caesium chloride (CsCl) density gradient centrifugation, was determined by qPCR (middle and bottom). (**b**) Replication of the subtelomeric late origins is affected in *sgo2*Δ cells. The strains used in **a** were released from G_2_/M arrest and their DNA was labelled with BrdU for 90 min in the presence of hydroxyurea (HU; 10 mM). The BrdU-labelled DNA was purified by immunoprecipitation and quantified by quantitative PCR. Each value was normalized to that of *ars2004*. Error bars indicate the s.d. (*n*=3). Asterisks indicate a significant change as compared with the wt strain (*P*<0.05, two-tailed *t*-test). (**c**) Replication of *sgo2*Δ, *bub1-KD* (kinase-dead) and *h2a-S121* mutants in the presence of HU. The experiments were performed as in **b**. Error bars indicate the s.d. (left, *n*=4; right, *n*=3). Asterisks indicate a significant change as compared with the wt strain (*P*<0.05, two-tailed *t*-test). (**d**) Recruitment of Mcm6 and Sld3 to the replication origins. *cdc25-22 nda4-108* mutant cells were released from G_2_/M arrest and then incubated for 180 min at 20 °C. ChIP analyses using anti-Mcm6 and anti-Flag (for Sld3-Flag) antibodies were performed. Each value was normalized to that of *non-ori*. Error bars indicate the s.d. (*n*=3). Asterisks indicate a significant change as compared with the wt strain (*P*<0.05, two-tailed *t*-test).

First, we analysed the replication kinetics at various origins on chromosome 2 in wt and *sgo2*Δ cells (Fig. 7a top). Cells were arrested at the G_2_/M boundary and released in the presence of a heavy-density analogue of thymidine, 5-bromo-2′-deoxyuridine (BrdU), which labels newly synthesized DNA in the following S phase; the incorporation of BrdU was then quantified to evaluate replication. In wt cells, *AT2088* (internal late origin) and *non-ori* (non-origin region) replicated ∼10 min later than *ars2004* (early origin), whereas the ST late origin *tel-21.2* replicated last among all of the locations examined (Fig. 7a middle and Supplementary Fig. 11 upper). Strikingly, in *sgo2*Δ cells, *tel-21.2* replicated with a similar or slightly earlier timing than *AT2088* and *non-ori* (Fig. 7a bottom and Supplementary Fig. 11 lower).

We also examined replication during early S phase by treating cells with hydroxyurea, which depletes the pools of deoxynucleoside triphosphate and thereby restricts further replication after its initiation at the early origins. In wt cells, *AT2088* and the ST late origins at *subtel2R* did not replicate efficiently as *ars2004* (Fig. 7b). In *sgo2*Δ cells, however, the ST late origins replicated with greater efficiency than observed in wt cells, whereas the replication level at *AT2088* did not increase (Fig. 7b). Furthermore, the increase in the replication levels in *sgo2*Δ cells involved 11.8–94.1 kb at *subtel2R*, which corresponds to the region of Sgo2 occupancy (Figs 1c and 7b). Indeed, as in *sgo2*Δ cells, replication at the subtelomeres was accelerated in the *bub1-KD* and *h2a-S121A* mutants, in which Sgo2 was removed from the subtelomeres (Fig. 7c and Supplementary Fig. 6). These results indicate that the association of Sgo2 with the subtelomeres has a repressive effect on replication to maintain proper replication timing at the ST late origins.

### Sgo2 limits loading of Sld3 to the replication origins

Next, we explored which step of replication is regulated by Sgo2. The initiation of DNA replication requires the formation of a pre-replicative complex (pre-RC) at origins during G_1_ phase, followed by activation of the pre-RC on entry into S phase. We therefore examined the loading of two replication factors, Mcm6 and Sld3, in cells arrested immediately before the stage of replication initiation^42^.

Mcm6, a pre-RC component, was enriched at *AT2088* and at the ST late origins (*tel-45.7* or *tel-21.2*) as efficiently as at *ars2004* in both wt and *sgo2*Δ cells (Fig. 7d left). In contrast, Sld3, which is recruited to origins during the earliest step of pre-RC activation on S phase entry^43^, was enriched at *ars2004* but not at the late origins in wt cells, as previously reported^41^ (Fig. 7d right). In *sgo2*Δ cells, recruitment of Sld3 was accelerated at *tel-45.7* and *tel-21.2*, but not at the internal late origin *AT2088* (Fig. 7d right). These data suggest that Sgo2 represses the initiation step of replication by limiting Sld3 loading at the ST late origins, which is required for proper replication timing.

## Discussion

**Figure 8:**
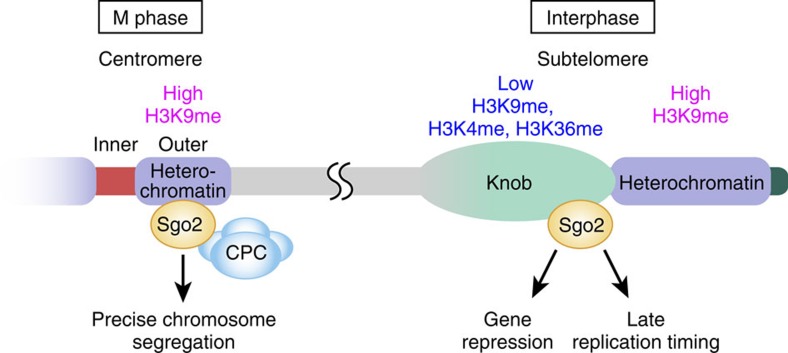
Model of the multiple roles of Sgo2 during the cell cycle. During M phase, Sgo2 accumulates at the outer regions of the centromeres, where H3K9me is enriched and recruits CPC for precise chromosome segregation. In interphase, Sgo2 is enriched at the heterochromatin-adjacent region of the subtelomeres, where the levels of the histone modifications such as H3K9me, H3K4me and H3K36me are relatively low. Sgo2 is essential to form the knob structures and to repress both gene transcription and replication, to ensure proper gene expression and replication timing.

In this study, we have discovered new regulations and functions of the subtelomere. We have shown that Sgo2, which accumulates at the centromeres and contributes to precise chromosome segregation during M phase, is recruited to the subtelomeres during interphase, especially G_2_ phase, and plays a role in the organization of the condensed chromatin body called a ‘knob', in the repression of gene transcription and in the repression of replication, to ensure proper gene expression and replication timing at the subtelomeres (Fig. 8).

It is currently unknown how Sgo2 is targeted to the subtelomeres during interphase. Phosphorylation of H2A-S121 can be observed outside the subtelomeres in interphase (Supplementary Fig. 4), indicating that H2A-S121 phosphorylation alone is not sufficient, and that another factor is required to restrict Sgo2 access to the subtelomeres. One possibility is that Teb1—a regulator of histone gene expression that localizes at the subtelomeres^44^—may support the recruitment of Sgo2. As Sgo2 dynamically changes its location during the cell cycle, some cell cycle regulators may also be involved in Sgo2 recruitment to the subtelomeres.

Notably, knobs are not formed at the centromeres at any stage of the cell cycle^34^, despite the high accumulation of Sgo2 at that location in M phase, suggesting that Sgo2 requires other factors to form knobs. The detailed mechanism underlying the subtelomere-specific formation of knobs remains unclear; however, one possibility is that ST chromatin contributes to this process. ST chromatin is characterized by its unique histone composition with low levels of acetylated and methylated histones, and the histone H2A variant H2A.Z^11^,^45^. The low level of H3K9 methylation at the telomere-distal region of the subtelomeres (adjacent to the ST heterochromatin region) is in marked contrast to the high level of H3K9 methylation at the Sgo2-associated outer regions of the centromeres^11^,^18^. In fact, knobs are observed preferentially at the telomere-distal regions of the subtelomeres^34^ (Fig. 4b).

Set2 is important for Sgo2 localization and knob formation^34^ (Fig. 4c,d); however, the level of Set2-dependent H3K36 methylation was found to be increased by Sgo2 deletion (Fig. 5e), which might seem to be contradictory. H3K36 methylation has been shown to be associated with transcription elongation and is observed at actively transcribed regions^35^,^36^. Therefore, it is likely to be that Set2 recruited by the transcription at subtelomeres somehow promotes Sgo2 localization, thereby leading to knob formation. Either the knob or Sgo2 itself may in turn repress the transcription and/or accessibility of histone-modifying enzymes including Set2, thereby forming a negative feedback loop. Removal of Sgo2 from the subtelomeres would resolve the condensed knob structures, leading to the activation of gene expression and the recruitment of Set2, resulting in the observed increase in H3K36 methylation.

Does the knob structure itself regulate gene transcription and replication? The highly condensed nature of a knob may limit access of the transcription and replication machineries to chromatin, which would be consistent with the observation that the association of both RNAPII and the replication regulator Sld3 with the subtelomeres was increased in knob-deficient *sgo2*Δ cells (Figs 5d and 7d). Another possibility is that Sgo2 itself recruits some regulatory factors to the subtelomeres. Recent studies in *S. cerevisiase* have revealed that the telomere-binding protein Rif1 regulates the replication timing of late origins by interacting with PP1 phosphatase to counteract the action of Dbf4-dependent kinase at the initiation step of replication^46^,^47^,^48^. Interestingly, Sgo1 forms a complex with PP2A phosphatase to protect cohesin in *S. pombe*, *S. cerevisiae* and mammals^21^,^49^,^50^,^51^, and human Sgo2 also interacts with PP2A for the protection of cohesion^52^. Thus, *S. pombe* Sgo2 may also interact with a protein phosphatase to counteract with the activity of Dbf4-dependent kinase and thereby regulate Sld3 loading.

It has been predicted that early replication from the subtelomere region will lead to an increase in the length of telomere DNA^53^,^54^. We therefore checked whether the early replication caused by Sgo2 deletion leads to abnormal telomere elongation. Telomere Southern analysis showed that the telomere DNA lengths were only slightly shorter in *sgo2*Δ and *bub1*Δ cells than in wt cells (Supplementary Fig. 12), suggesting that Sgo2 is not important for telomere length control.

What are the physiological roles of the ST gene repression caused by Sgo2? Matsuda *et al*.^34^ reported that the frequency of knob formation substantially decreased when cells were arrested in G_1_ phase by nitrogen starvation. In this condition, Sgo2 might dissociate from the subtelomeres, inducing the expression of some ST genes in response to the nutrient stress. Another stress condition—low glucose stress (from 111 to 4.4 mM)—has been shown to induce the expression of a set of hexose transporter genes called *ght1*^+^*-ght8*^+^ (ref. 55), of which *ght3*^+^ and *ght7*^+^ are located at the subtelomeres. As shown in Fig. 5c, deletion of Sgo2 caused an increase in the expression of *ght3*^+^ and *ght7*^+^. We speculate that Sgo2 might dissociate from the subtelomeres under some stress conditions including low glucose, to enable the efficient activation of ST gene expression for cell survival.

In summary, this study has revealed that Sgo defines chromatin functions not only at the centromeres but also at the subtelomeres in a cell cycle-dependent manner. Thus far, ST localization of Sgo in other organisms has not been reported; however, some cases in which Sgo relocates from the centromeres to the chromosome arms have been demonstrated in human cells and in *Xenopus* egg extracts^56^,^57^. Thus, one can speculate that the translocation of Sgo to subtelomeres or other chromosomal regions might lead to the local organization of a repressive chromatin domain in other eukaryotes, as it does in *S. pombe*.

## Methods

### *S. pombe* strains

All of the experiments were performed using the *S. pombe* strains listed in Supplementary Table 1. Growth media, basic genetics and construction of strains carrying deletion alleles or epitope-tagged proteins were described previously^58^,^59^,^60^. The primers listed in Supplementary Table 2 were used to generate DNA fragments for homologous recombination to produce the deletion and carboxy-terminal epitope-tagged strains^59^,^61^.

### Calculating the distance from a telomere

We determined the distance from the telomeric repeat DNA on the basis of the following assumptions:

- The sequence between contig PJ5566 (ref. 7) and *tel2R* is the same as that in the pNSU70 vector^6^. This assumption provides the complete *subtel2R* sequence.
- Other unsequenced regions of the subtelomeres, such as the region between *tel1L* and contig c212, a gap between contigs c212 and c977, a region between contig c977 and *tel1R*, and a region between *tel2L* and contig c1348, have the same sequences as their *subtel2R* counterparts. This assumption is valid because of the high degree of sequence similarity among the subtelomeres^7^. The sequence of the gap between contigs c1348 and pB10D8 has been reported previously^62^.

On the basis of these assumptions, we obtained the following equations for calculating the distances from telomeres:


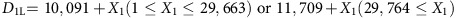



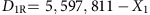



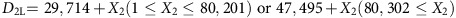



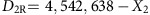


where *X*_1_ or *X*_2_ is the chromosome position number on chromosome 1 or 2, respectively, and *D*_1L_, *D*_1R_, *D*_2L_ or *D*_2R_ is the calculated distance between *X*_1_ and *tel1L*, between *X*_1_ and *tel1R*, between *X*_2_ and *tel2L*, or between *X*_2_ and *tel2R*, respectively.

### ChIP assays

Exponentially growing cells were fixed at 32 °C for 30 min in 3% paraformaldehyde (for Sgo2-Flag detection) or 1% formaldehyde (for histone modification and RNAPII detection). For the experiments in Fig. 2a (lower) and Supplementary Fig. 7, cells grown in YPD liquid medium at 32 °C were transferred to 20 °C, incubated for 12 h and then fixed for 30 min in 3% paraformaldehyde. For those in Fig. 2b, temperature-sensitive *cdc25-22* mutant cells were grown in YES liquid medium to log phase (2.5 × 10^6^ cells per ml) at the permissive temperature (25 °C) and then incubated at the restrictive temperature (35.5 °C) for 4 h for cell-cycle arrest at the G_2_/M boundary. Next, the culture was transferred to 25 °C for cell cycle release and a portion was fixed with 3% paraformaldehyde at each time point. Crude extracts were prepared by breaking the fixed cells in lysis buffer (50 mM HEPES-KOH pH 7.5, 140 mM NaCl, 1 mM EDTA, 1% Triton X-100, 0.1% sodium deoxycholate, 1 × cOmplete (Roche) and 1 mM phenylmethylsulfonyl fluoride) using a Multi-beads Shocker (Yasui Kikai), after which the chromatin was sheared by sonication using Bioruptor UCD-250 (BM Equipment) to an average DNA length of 0.5 kb. As shown in Supplementary Fig. 5, a portion of each sonicated crude extract was used for immunoblotting. The insoluble fraction of the crude extract was removed by centrifugation and the supernatant was used for immunoprecipitation (see Antibodies) using Dynabeads M-280 anti-Mouse IgG or anti-Rabbit IgG (Thermo Fisher) as a carrier. The beads were washed once with Buffer 1 (50 mM HEPES-KOH pH 7.5, 140 mM NaCl, 1 mM EDTA, 1% Triton X-100 and 0.1% sodium deoxycholate), once with Buffer 1′ (50 mM HEPES-KOH pH 7.5, 500 mM NaCl, 1 mM EDTA, 1% Triton X-100 and 0.1% sodium deoxycholate), once with Buffer 2 (10 mM Tris-HCl pH 8.0, 250 mM LiCl, 1 mM EDTA, 0.5% NP-40 and 0.5% sodium deoxycholate) and twice with TE buffer (10 mM Tris-HCl pH 8.0 and 1 mM EDTA). Immunoprecipitants and input extracts were treated with 10 μg ml^−1^ RNase A in TE buffer for 30 min at 37 °C, then with 250 μg ml^−1^ proteinase K in 0.25% sodium lauryl sulfate (SDS; 37 °C overnight), followed by the reverse cross-linking reaction (65 °C for 6 h). Associated DNA fragments were purified by phenol chloroform extraction and ethanol precipitation, and then analysed by quantitative PCR (qPCR) using a StepOne real-time PCR system. The sequences of the primer sets used for real-time PCR are listed in Supplementary Table 3.

### ChIP–chip analyses

DNA labelling, microarray slide hybridization (custom array GPL7277, Agilent Technologies)^63^, microarray visualization and data extraction were performed as described previously^64^.

### Microscopy

For the experiments in Fig. 1e and Supplementary Fig. 2, cells were exponentially grown in the EMM medium at 30 °C and 13 optical section images were obtained at focus intervals of 0.3 μm using a DeltaVision microscopy system (Applied Precision) and deconvolved by a three-dimensional deconvolution method using SOFTWORX software^65^.

### Knob observation

Cells exponentially growing in EMM2-5S at 26 °C were pelleted by gentle centrifugation, chemically fixed by re-suspension in a buffer containing 4% formaldehyde (Polysciences, Inc.), 80 mM HEPES-K, 35 mM HEPES-Na, 2 mM EDTA, 0.5 mM EGTA, 0.5 mM spermidine, 0.2 mM spermine and 15 mM 2-mercaptethanol pH 7.0, for 10 min at room temperature, and then washed three times in PEMS (100 mM PIPES, 1 mM EGTA, 1 mM MgSO_4_, 1.2 M sorbitol pH 6.9) with 2–5 min of rotation. The cell wall was digested by incubation with 0.6 mg ml^−1^ of zymolyase 100 T (Seikagaku Biobusiness) in PEMS at 36 °C for 5–30 min. The cells were then treated with 0.1% TritonX-100 in PEMS for 5 min and washed three times with PEMS.

For DAPI staining, the cells were incubated with 0.2 μg ml^−1^ of DAPI for 10 min and mounted with nPG-Glycerol (100% glycerol for absorptionmetric-analysis (Wako) with 4% n-propyl gallate, pH 7.0). For immunostaining, the cells were incubated with 1% BSA, 0.1% NaN_3_, 100 mM L-lysine monohydrochloride in PEMS (PEMSBAL) for 30 min; the solution was then replaced with antibody in PEMSBAL and stained for 1 h. The cells were washed three times with PEMSBAL and then secondary antibody was added. Afterwards, the cells were washed, counterstained with DAPI and suspended in mounting medium (nPG-Glycerol). DeltaVision|OMX microscope version 3 (GE Healthcare) was used for three-dimensional structured illumination microscopy (3DSIM) imaging with an objective lens, × 100 UPlanSApo NA1.40 Oil (Olympus). Conspicuously condensed, DAPI-stained bodies were counted as ‘knobs' by visually inspecting each optical section of the 3DSIM images.

### Antibodies

For ChIP, immunostaining and western blot analyses, we used the following antibodies: monoclonal anti-Flag M2 (Sigma, F3165; 1:4,200 dilution for ChIP in Figs 1, 2, 3, 4 and Supplementary Figs 3 and 7; 1:8,000 for western blotting), monoclonal anti-Flag M2 (Sigma, F1804; 1:500 for ChIP in Fig. 7 and Supplementary Fig. 6), anti-H2AS121ph^22^ (1:500 for ChIP), anti-GFP (Roche, 11814460001; 1:500 for ChIP), anti-RNAPII clone CTD4H8 (Millipore, 05-623; 1:250 for ChIP), anti-H3K36me3 (MAB Institute, MABI0333; 1:1,000 for ChIP), anti-H3K4me2 (MAB Institute, MABI0303; 1:1,000 for ChIP), anti-BrdU (Becton Dickinson, 347580; 0.5 μg per reaction) and anti-Mcm6 (ref. 66; 1:500 for ChIP). Anti-H3K9me3 (CMA318 (2F3)^67^; 1:500) and anti-GFP (MBL, 598; 1:500) antibodies were used in the observation of knobs.

### RNA quantification

Exponentially growing cells in YES liquid medium were harvested by centrifugation and washed once with double distilled water. Total RNA was prepared by breaking cells in water-saturated phenol and RNA extraction buffer (20 mM Tris-HCl pH 7.5, 300 mM NaCl, 10 mM EDTA and 1% SDS) using a Multi-beads Shocker (Yasui Kikai). The total RNA was further purified by 0.2 U μl^−1^ recombinant DNase I (RNase-free) (TaKaRa Bio) treatment (37 °C for 30 min) followed by phenol chloroform extraction and ethanol precipitation. Complementary DNAs were synthesized from total RNA using the High-Capacity cDNA Reverse Transcription Kit (Applied Biosystems) and then analysed by qPCR using a StepOne real-time PCR system. The sequences of the primer sets corresponding to each gene body used for real-time PCR are listed in Supplementary Table 3.

### Microarray analysis

Poly(A)-containing RNA was purified from total RNA using a PolyATtract mRNA Isolation System (Promega). The biotin-labelled cRNAs were synthesized from 0.3 μg of messenger RNA using GeneChip One-Cycle Target Labeling and Control Reagents (Affymetrix) and were hybridized to a GeneChip Yeast Genome 2.0 Array (Affymetrix) in accordance with the manufacturer's instructions. After staining, the GeneChips were scanned using GeneChip Scanner 3000 G7 and GeneChip Operating Software (GCOS 1.4). The scaling and comparative analyses were performed by using GCOS 1.4 software with the default settings and *S. cerevisiae* probe set masking.

### Replication timing analyses

Chromosome DNA replication was analysed by BrdU incorporation assays as described previously^39^,^68^ with some modifications. Derivatives of *cdc25-22 Pnmt1-TK Padh1-hENT* expressing the herpes simplex virus thymidine kinase gene and the human equilibrative nucleoside transporter gene, respectively, were incubated at 36 °C for 3 h for G_2_/M arrest and then released at 25 °C in the presence of 200 μM BrdU (Sigma). Cells collected at the indicated time were fixed with 0.1% sodium azide and total cellular DNA was prepared by the smash and grab method^69^. For replication kinetics analysis, the cellular DNA was digested with HaeIII, centrifuged in a CsCl density gradient to separate the replicated DNA (heavy–light (HL) density) from unreplicated DNA (light–light (LL) density) and the amount of DNA in the HL and LL densities was determined by qPCR using a 7300 real-time PCR System (Applied Biosystems). The primers used for qPCR are listed in Supplementary Table 3. Replication (%) was calculated with the equation: (1/2HL)/(1/2HL+LL). To determine the extent of replication in early S phase, 10 mM hydroxyurea (Sigma) and 200 μM BrdU were added to the cells on release from the G_2_/M block. The genomic DNA was sonicated into fragments of around 400 bp, denatured by boiling and used for immunoprecipitation with anti-BrdU antibody associated with Dynabeads M-280 anti-Mouse IgG (Thermo Fisher). The immunoprecipitated DNA was recovered and purified as described in the Methods for ChIP, except that there was no reverse cross-linking. The immunoprecipitated DNA and the input DNA were analysed by qPCR. For ChIP analyses of Mcm6 and Sld3-Flag, wt and *sgo2*Δ cells carrying *cdc25-22 nda4-108/mcm5 sld3-5flag* were synchronized at the G_2_/M boundary and released at 20 °C (the restrictive temperature for *nda4-108*) for 180 min, allowing Sld3 loading without the initiation of DNA synthesis. The soluble cell extracts were used for ChIP analysis with anti-Mcm6 and anti-Flag (for Sld3-Flag) antibodies. The immunoprecipitated DNA and the input DNA were analysed by qPCR.

### Pulsed-field gel electrophoresis analysis

Intact chromosomes in an agarose plug were electrophoresed using CHEF-DR III Pulsed Field Electrophoresis Systems (Bio-Rad) under the following conditions: 0.8% SeaKem Gold Agarose (Lonza) in 1 × TAE; temperature, 14 °C; Block 1: initial switch time 1,200 s, final switch time 1,200 s, run time 24 h, voltage gradient 2 V cm^−1^, angle 96°; Block 2: initial switch time 1,500 s, final switch time 1,500 s, run time 24 h, voltage gradient 2 V cm^−1^, angle 100°; Block 3: initial switch time 1,800 s, final switch time 1,800 s, run time 24 h, voltage gradient 2 V cm^−1^, angle 106°. Chromosomal DNA separated in the gel was transferred to a Hybond-N^+^ (GE Healthcare) membrane according to the manufacturer's instructions. To generate the probes, the TAS fragments (TAS1, TAS2 and TAS3)^29^ were excised from pNSU70 (ref. 6) and an rDNA fragment was amplified by PCR from *S. pombe* genomic DNA using the following primer set:

st144: 5′-CGCTAACCATTATTTACTGAGGAGAAC-3′

st160: 5′-CAAATGTAACCCTCATTAAAAATATTAC-3′

In Southern blot analysis, these DNA fragments were labelled with digoxigenin using the DIG High Prime DNA Labelling and Detection Starter Kit II (Roche).

## Additional information

**Accession codes:** ChIP–chip and microarray data are available in the Gene Expression Omnibus database under accession codes GSE61699 and GSE61698, respectively.

**How to cite this article:** Tashiro, S. *et al*. Shugoshin forms a specialized chromatin domain at subtelomeres that regulates transcription and replication timing. *Nat. Commun.* 7:10393 doi: 10.1038/ncomms10393 (2016).

## Supplementary Material

Supplementary Information Supplementary Figures 1-12, Supplementary Tables 1-3 and Supplementary References
