# Supplementary material for: Shugoshin forms a specialized chromatin domain at subtelomeres that regulates transcription and replication timing
Source: Nat Commun. 2016 Jan 25;7:10393. doi: 10.1038/ncomms10393 (PMC4737732; doi:10.1038/ncomms10393)
Supplement: Supplementary Information — Supplementary Figures 1-12, Supplementary Tables 1-3 and Supplementary References [file ncomms10393-s1.pdf]

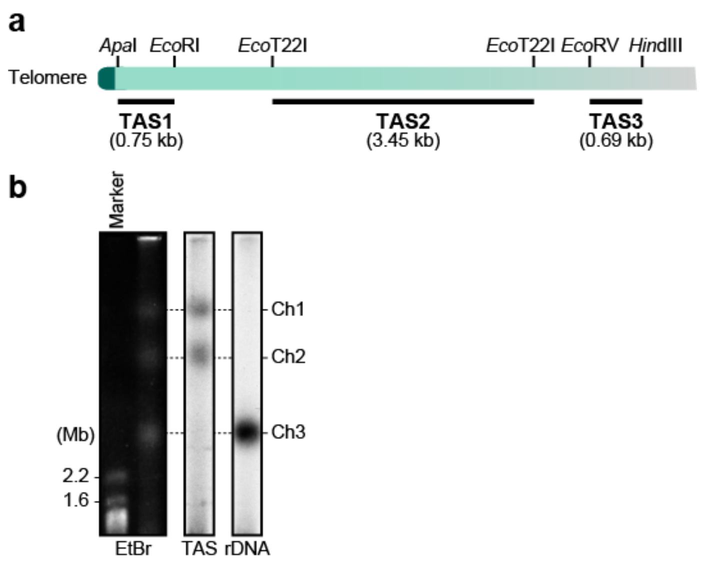

Supplementary Figure 1. Chromosome 3 is devoid of the telomere-proximal subtelomeric common sequences.

(a) Schematic illustration of the telomere-proximal site of subtelomeres. The restriction map is based on the previously cloned telomere-containing plasmid pNSU70[1].

(b) Pulsed-field gel electrophoresis of the intact three chromosomes of the *S. pombe* wild-type strain, *972 h*$^-$ followed by Southern blot analysis using TAS (a mixture of TAS1, TAS2, and TAS3)[2] or rDNA as probes. *S. cerevisiae* chromosomes were used as size markers.

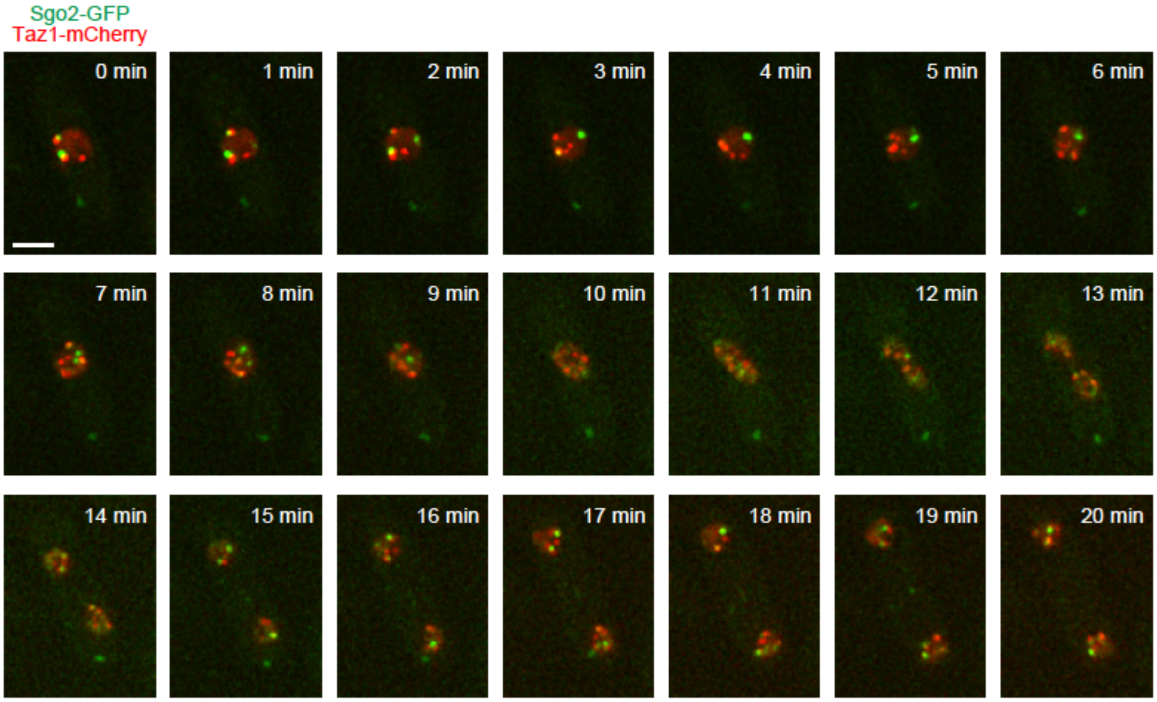

Supplementary Figure 2. Subnuclear localization of Sgo2 in a living cell.

Three-dimensional deconvolved time-lapse images of a single cell grown in EMM at 30°C. Each image is a projection of three-dimensional optical sections. The green signals represent Sgo2-GFP. The red signals represent Taz1-mCherry (telomeres). Sgo2-GFP signals were located near the telomeres at time 0 (the start point of the observation) and began to dissociate from the telomeres at 1 min. Sgo2-GFP was relocated to the vicinity of the telomeres after nuclear division at 13 min. Scale bar, 2 µm.

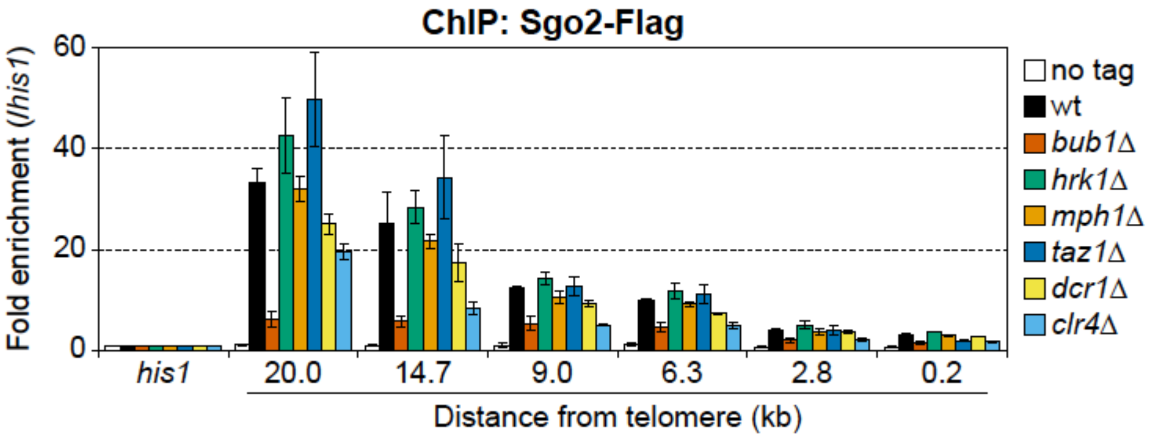

Supplementary Figure 3. Bub1 is important for localization of Sgo2 at the telomere-proximal subtelomeric region.

ChIP analyses of Sgo2-Flag localization at the telomere-proximal subtelomeric region in various mutants. Relative fold enrichment, normalized to the signal at the *his1*$^{+}$ locus, is shown. Error bars indicate the SD (n = 3).

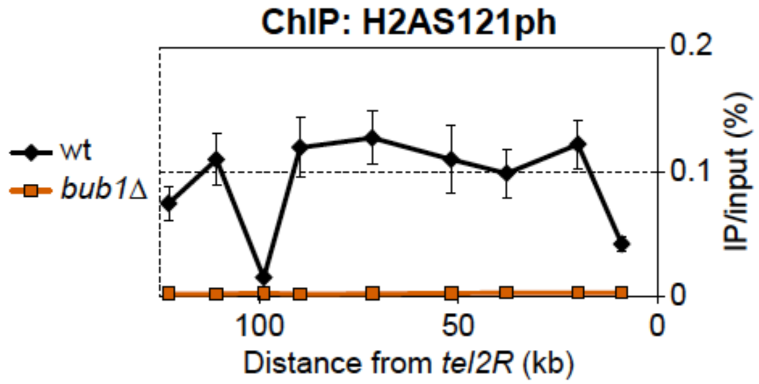

Supplementary Figure 4. Lack of H2A-S121 phosphorylation in *bub1*Δ cells.

ChIP analyses of phosphorylated H2A-S121 in wild-type and *bub1*Δ cells. The recovery of immunoprecipitated DNA relative to total input DNA was measured by quantitative PCR. Error bars indicate the SD (n = 3).

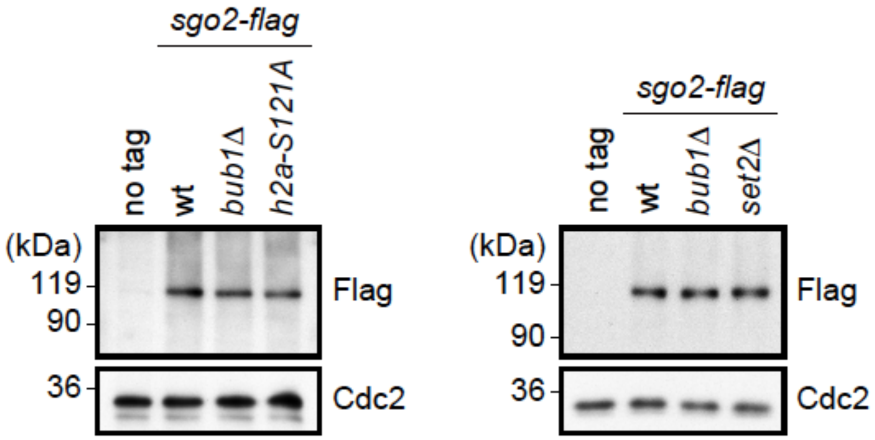

Supplementary Figure 5. Sgo2-Flag protein levels.

Western blot analysis of Sgo2-Flag protein levels in the strains used in Figs. 3c (left) and 4d (right). Cdc2 was used as a loading control.

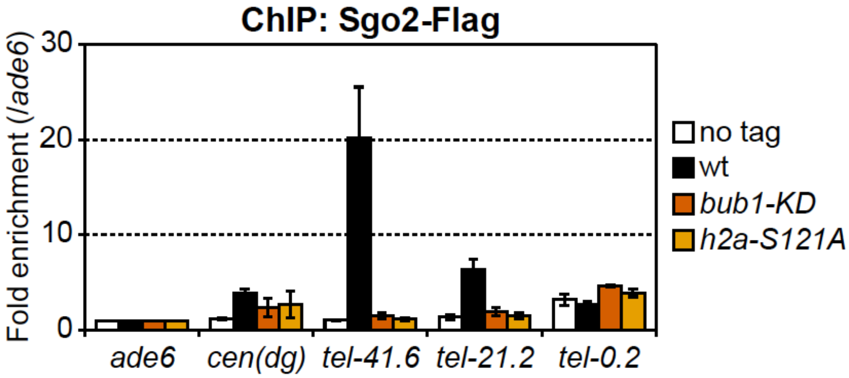

Supplementary Figure 6. Kinase activity of Bub1 is required for the subtelomeric localization of Sgo2.

ChIP analyses of Sgo2-Flag localization in *bub1-KD* (kinase-dead) and *h2a-S121A* mutant cells. Relative fold enrichment at the centromeres (*dg*) and subtelomere loci (*tel-41.6*, *tel-21.2*, and *tel-0.2*), normalized to the signal at the $ade6^{+}$ locus, is shown. No tag indicates the negative control for the ChIP analysis. Error bars indicate the SD (n = 3).

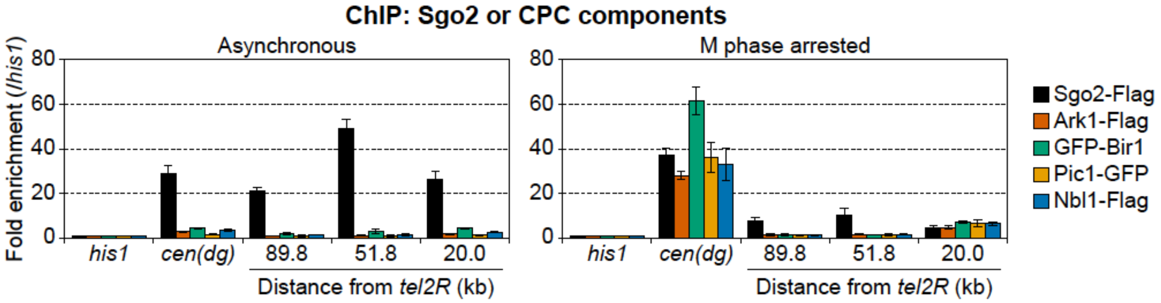

Supplementary Figure 7. CPC components are barely detected at the subtelomeres during interphase.

ChIP analyses of the localization of Sgo2 and CPC components. Cold-sensitive *nda3-KM311* mutant cells were grown at 32°C (asynchronous cells, predominantly in interphase, left) or 20°C for 12 h (M phase-arrested, right) in YPD medium. Relative fold enrichment at *subtel2R*, normalized to the signal at the *his1*$^+$ locus, is shown. *cen* (*dg*) was a positive control for localization of the CPC components. Error bars indicate the SD (*n* = 3).

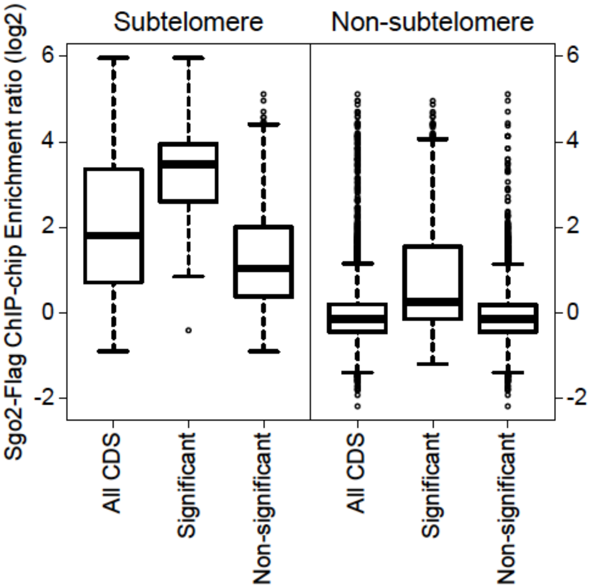

Supplementary Figure 8. Correlation between Sgo2-dependent gene regulation and the amount of subtelomeric Sgo2.

The box plots represent the distribution of the Sgo2-Flag ChIP–chip signals at subtelomeric or non-subtelomeric genes (shown in Fig. 1b), whose expression was significantly affected or not by Sgo2 deletion (shown in Fig. 5a). ChIP–chip array probes that overlapped with the coding DNA sequence (CDS) regions on the expression array were extracted. The box plots summarize the distributions of the ChIP–chip enrichment ratio of the extracted probes. Sgo2 is enriched at the subtelomeric CDS regions, particularly in significant CDS regions, as compared with the non-subtelomeric CDS regions.

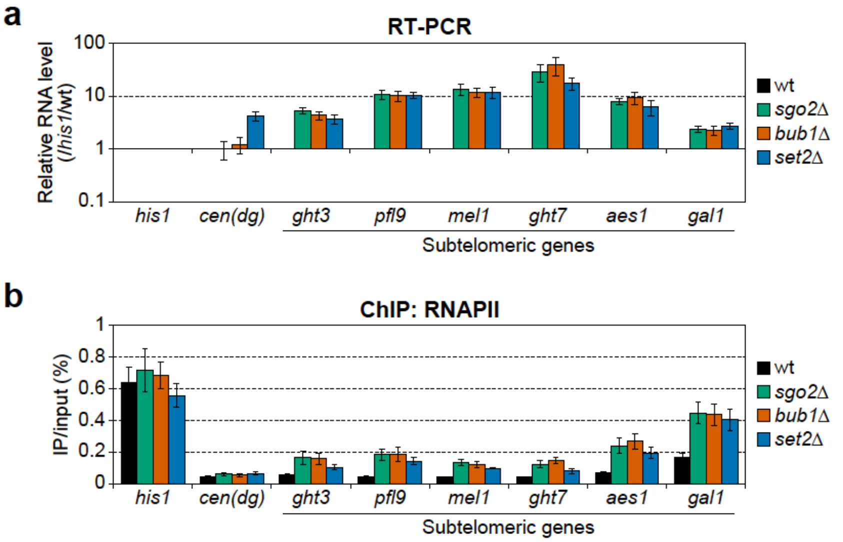

Gene	Distance from telomere
ght3	94.7 kb (*tel1L*)
pfl9	70.1 kb (*tel1R*)
mel1	97.3 kb (*tel1R*)
ght7	69.0 kb (*tel2L*)
aes1	100.6 kb (*tel2L*)
gal1	53.5 kb (*tel2R*)

Supplementary Figure 9. Set2 is important for gene repression at the subtelomeres.

(a) The RNA expression levels of the subtelomeric genes and the centromeric repeats (*dg*) in wild-type, *sgo2*Δ, *bub1*Δ, and *set2*Δ cells were analyzed by quantitative RT-PCR. Each value was normalized to that of *his1*$^{+}$, and then re-normalized to the wild-type value.

(b) The rate of occupancy of RNAPII at the subtelomeric gene loci increased in *sgo2*Δ, *bub1*Δ, and *set2*Δ cells. ChIP analyses of the RNAPII levels at the subtelomeric gene loci and at the centromeric repeats (*dg*). The recovery of immunoprecipitated DNA relative to total input DNA was measured by quantitative PCR. Error bars indicate the SD (n = 3).

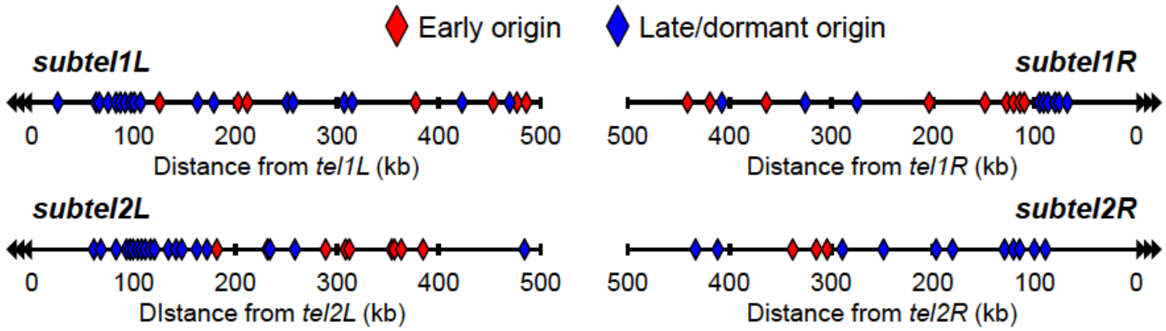

Supplementary Figure 10. Map of the early and late replication origins near the telomeres.

The positions of the early (red) and late (blue) origins near the telomeres identified previously[3,4] are shown with modification.

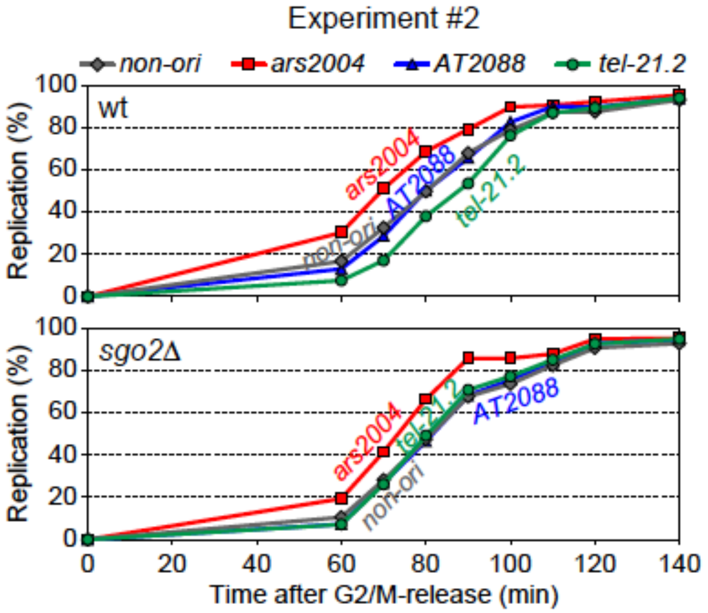

Supplementary Figure 11. Independent experimental data confirming the reproducibility of Figure 7a.

The experiment was performed as described in Figure 7a.

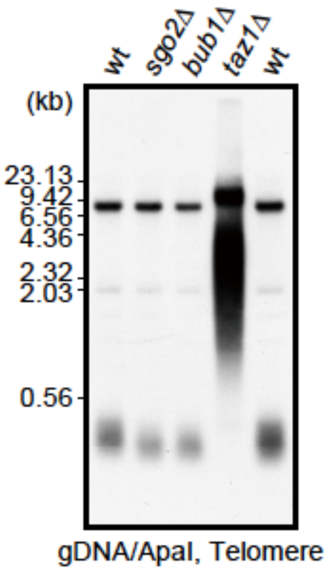

Supplementary Figure 12. Telomere DNA length in *sgo2*Δ and *bub1*Δ cells.

Southern blot analyses of *Apa*I-digested genomic DNA for telomere DNA length. Telomere repeat DNA was used as a probe.

Supplementary Table 1. *S. pombe* strains used in this study

**Fig. 1b, c**	
KM672	*h*$^-$ *leu1-32 ura4-D18 sgo2-3flag-kan*^{r}
**Fig. 1d**	
JK107	*h*$^-$
ST2915	*h*$^-$ *sgo2-3flag-kan*^{r}
**Fig. 1e**	
JP2989	*h*$^-$ *leu1-32 ura4-D18 taz1-mcherry-hyg*^{r} *sgo2-gfp-kan*^{r} *gar1-cerulean-nat*^{r}
**Fig. 2a**	
JK107	*h*$^-$
ST2915	*h*$^-$ *sgo2-3flag-kan*^{r}
JP776	*h*$^-$ *leu1 ura4 nda3-KM311 sgo2-3flag-kan*^{r}
**Fig. 2b**	
ST4229	*h*$^-$ *leu1-32 ura4-D18 cdc25-22 sgo2-3flag-kan*^{r}
**Fig. 3a**	
KM1399	*h*^{90} *ade6-M210 leu1-32 ura4-D18 sgo2-3flag-kan*^{r}
KM1615	*h*^{90} *ade6-M210 leu1-32 ura4-D18 trt1*::*LEU2* (Type A) *sgo2-3flag-kan*^{r}
KM1618	*h*^{90} *ade6-M210 leu1-32 ura4-D18 trt1*::*LEU2* (Type B) *sgo2-3flag-kan*^{r}
**Fig. 3b**	
JK107	*h*$^-$
ST2915	*h*$^-$ *sgo2-3flag-kan*^{r}
JP3834	*h*$^-$ *bub1*::*hyg*^{r} *sgo2-3flag-kan*^{r}
TB3417	*h*$^-$ *sgo2-3flag-kan*^{r} *hrk1*::*hyg*^{r}
JP3838	*h*$^-$ *sgo2-3flag-kan*^{r} *mph1*::*hyg*^{r}
JP3836	*h*$^-$ *taz1*::*kan*^{r} *sgo2-3flag-hyg*^{r}
JP3829	*h*$^-$ *sgo2-3flag-kan*^{r} *dcr1*::*hyg*^{r}
JP3844	*h*$^-$ *clr4*::*kan*^{r} *sgo2-3flag-hyg*^{r}
**Fig. 3c**	
TB3448	*h*$^-$ *ade6-M216 leu1-32 sgo2-3flag-hyg*^{r}
JP3906	*h*$^-$ *ade6-M216 leu1-32 sgo2-3flag-hyg*^{r} *bub1*::*kan*^{r}

TB3450 — h^- *ade6-M216 leu1-32 hta1(S121A) hta2(S121A) sgo2-3flag-*hyg^r

Fig. 4a

YAM236 — h^{90} *sgo2-gfp-*hyg^r

Fig. 4b

YAM055 — h^- *taz1-gfp-*kan^r

YAM198 — h^- *ade6-216 sgo2::*kan^r *taz1-gfp-*kan^r

Fig. 4c

972 — h^-

PZ876 — h^- *ade6-216 leu1 sgo2::*kan^r

AY356-5D — h^- *leu1 bub1::*kan^r

YAM081 — h^+ *his2 leu1 lys1 ade6-216 set2::*kan^r

JP3415 — h^- *ade6-M216 leu1-32*

JP3416 — h^- *ade6-M216 leu1-32 hta1(S121A) hta2(S121A)*

Fig. 4d

ST2915 — h^- *sgo2-3flag-*kan^r

JP3834 — h^- *bub1::*hyg^r *sgo2-3flag-*kan^r

JP3850 — h^- *sgo2-3flag-*kan^r *set2::*hyg^r

Fig. 5a

JK107 — h^-

JP2247 — h^- *sgo2::*hyg^r

Fig. 5b

JK107 — h^-

JP2247 — h^- *sgo2::*hyg^r

KM672 — h^- *leu1-32 ura4-D18 sgo2-3flag-*kan^r

Fig. 5c, d, e

JP3415 — h^- *ade6-M216 leu1-32*

ST3888 — h^- *ade6-M216 leu1-32 sgo2::*kan^r

JP3914 — h^- *ade6-M216 leu1-32 bub1::*kan^r

JP3416 — h^- *ade6-M216 leu1-32 hta1(S121A) hta2(S121A)*

Fig. 6a

JK107	*h*$^-$
JP2247	*h*$^-$ *sgo2*::*hyg*^{r}
JK1330	*h*$^+$ *leu1-32 ura4-D18 T2R1-4137*:*ura4*$^+$
ST2944	*h*$^+$ *leu1-32 ura4-D18 T2R1-4137*:*ura4*$^+$ *sgo2*::*kan*^{r}
JK1390	*h*$^+$ *leu1-32 ura4-D18 T2R1-4137*:*ura4*$^+$ *swi6*::*LEU2*
JK1618	*h*$^-$ *leu1-32 ura4-D18 B2B2-18226*:*ura4*$^+$
ST2947	*h*$^-$ *leu1-32 ura4-D18 B2B2-18226*:*ura4*$^+$ *sgo2*::*kan*^{r}
JK1624	*h*$^-$ *leu1-32 ura4-D18 B2B2-18226*:*ura4*$^+$ *swi6*::*kan*^{r}

Fig. 6b

JK1618	*h*$^-$ *leu1-32 ura4-D18 B2B2-18226*:*ura4*$^+$
ST2947	*h*$^-$ *leu1-32 ura4-D18 B2B2-18226*:*ura4*$^+$ *sgo2*::*kan*^{r}
ST3892	*h*$^-$ *leu1-32 ura4-D18 B2B2-18226*:*ura4*$^+$ *bub1*::*kan*^{r}
ST3894	*h*$^-$ *leu1-32 ura4-D18 B2B2-18226*:*ura4*$^+$ *set2*::*kan*^{r}

Fig. 6c

JP2762	*h*$^-$ *leu1-32 ura4-D18*
JK107	*h*$^-$
JP2247	*h*$^-$ *sgo2*::*hyg*^{r}
JK1330	*h*$^+$ *leu1-32 ura4-D18 T2R1-4137*:*ura4*$^+$
ST2944	*h*$^+$ *leu1-32 ura4-D18 T2R1-4137*:*ura4*$^+$ *sgo2*::*kan*^{r}
JK1390	*h*$^+$ *leu1-32 ura4-D18 T2R1-4137*:*ura4*$^+$ *swi6*::*LEU2*
JK1618	*h*$^-$ *leu1-32 ura4-D18 B2B2-18226*:*ura4*$^+$
ST2947	*h*$^-$ *leu1-32 ura4-D18 B2B2-18226*:*ura4*$^+$ *sgo2*::*kan*^{r}
JK1624	*h*$^-$ *leu1-32 ura4-D18 B2B2-18226*:*ura4*$^+$ *swi6*::*kan*^{r}
ST3892	*h*$^-$ *leu1-32 ura4-D18 B2B2-18226*:*ura4*$^+$ *bub1*::*kan*^{r}
ST3894	*h*$^-$ *leu1-32 ura4-D18 B2B2-18226*:*ura4*$^+$ *set2*::*kan*^{r}

Fig. 7a, b

HM1864	*h*$^-$ *cdc25-22 ura4*::*ura4-Pnmt1-TK ade6*::*ade6-Padh1-hENT*
HM4771	*h*$^-$ *cdc25-22 ura4*::*ura4-Pnmt1-TK ade6*::*ade6-Padh1-hENT sgo2*::*hyg*^{r}

Fig. 7c

HM1864	*h*$^-$ *cdc25-22 ura4*::*ura4-Pnmt1-TK ade6*::*ade6-Padh1-hENT*
HM4771	*h*$^-$ *cdc25-22 ura4*::*ura4-Pnmt1-TK ade6*::*ade6-Padh1-hENT sgo2*::*hyg*^{r}
HM5313	*h*$^-$ *cdc25-22 bub1(K762R,D900N)*

ura4::ura4-Pnmt1-TK ade6::ade6-Padh1-hENT

HM5259 *h*$^-$ *cdc25-22 hta1(S121A) hta2(S121A) ura4::ura4-Pnmt1-TK ade6::ade6-Padh1-hENT*

Fig. 7d

HM2637 *h- cdc25-22 nda4-108 ura4::ura4-Pnmt1-TK sld3::sld3-5FLAG-kan*^{r}

HM5280 *h- cdc25-22 nda4-108 ura4::ura4-Pnmt1-TK sld3::sld3-5FLAG-kan*^{r} *sgo2::hyg*^{r}

Supplementary Fig. 1b

JK107 *h*$^-$

Supplementary Fig. 2

KM994 *h*$^-$ *leu1-32 ura4-D18 taz1-mcherry-hyg*^{r} *sgo2-gfp-kan*^{r}

Supplementary Fig. 3

JK107 *h*$^-$

ST2915 *h*$^-$ *sgo2-3flag-kan*^{r}

JP3834 *h*$^-$ *bub1::hyg*^{r} *sgo2-3flag-kan*^{r}

TB3417 *h*$^-$ *sgo2-3flag-kan*^{r} *hrk1::hyg*^{r}

JP3838 *h*$^-$ *sgo2-3flag-kan*^{r} *mph1::hyg*^{r}

JP3836 *h*$^-$ *taz1::kan*^{r} *sgo2-3flag-hyg*^{r}

JP3829 *h*$^-$ *sgo2-3flag-kan*^{r} *dcr1::hyg*^{r}

JP3844 *h*$^-$ *clr4::kan*^{r} *sgo2-3flag-hyg*^{r}

Supplementary Fig. 4

JK107 *h*$^-$

JP2251 *h*$^-$ *bub1::hyg*^{r}

Supplementary Fig. 5

JP3415 *h*$^-$ *ade6-M216 leu1-32*

TB3448 *h*$^-$ *ade6-M216 leu1-32 sgo2-3flag-hyg*^{r}

JP3906 *h*$^-$ *ade6-M216 leu1-32 sgo2-3flag-hyg*^{r} *bub1::kan*^{r}

TB3450 *h*$^-$ *ade6-M216 leu1-32 hta1(S121A) hta2(S121A) sgo2-3flag-hyg*^{r}

JK107 *h*$^-$

ST2915 *h*$^-$ *sgo2-3flag-kan*^{r}

JP3834 *h*$^-$ *bub1::hyg*^{r} *sgo2-3flag-kan*^{r}

JP3850 *h*$^-$ *sgo2-3flag-kan*^{r} *set2::hyg*^{r}

**Supplementary Fig. 6**	
HM4532	*h*$^-$
HM4846	*h*$^-$ *sgo2-6his3flag-hyg*^{r}
HM5242	*h*$^-$ *bub1(K762R,D900N) sgo2-6his3flag-hyg*^{r}
HM5236	*h*$^-$ *hta1(S121A) hta2(S121A) sgo2-6his3flag-hyg*^{r}
**Supplementary Fig. 7**	
JP776	*h*$^-$ *leu1 ura4 nda3-KM311 sgo2-3flag-kan*^{r}
JK195	*h*$^-$ *leu1 ura4 nda3-KM311 ark1-3flag-ura4*$^+$
KM1378	*h*$^-$ *leu1 ura4 nda3-KM311 gfp-bir1-ura4*$^+$
KM1226	*h*$^-$ *leu1 ura4 nda3-KM311 pic1-gfp-kan*^{r}
TB2635	*h*$^-$ *leu1 ura4 nda3-KM311 nbl1-3flag-kan*^{r}
**Supplementary Fig. 8**	
KM672	*h*$^-$ *leu1-32 ura4-D18 sgo2-3flag-kan*^{r}
JK107	*h*$^-$
JP2247	*h*$^-$ *sgo2*::*hyg*^{r}
**Supplementary Fig. 9**	
JK107	*h*$^-$
JP2247	*h*$^-$ *sgo2*::*hyg*^{r}
JP2251	*h*$^-$ *bub1*::*hyg*^{r}
JK3846	*h*$^-$ *set2*::*kan*^{r}
**Supplementary Fig. 11**	
HM1864	*h*$^-$ *cdc25-22 ura4*::*ura4-Pnmt1-TK ade6*::*ade6-Padh1-hENT*
HM4771	*h*$^-$ *cdc25-22 ura4*::*ura4-Pnmt1-TK ade6*::*ade6-Padh1-hENT sgo2*::*hyg*^{r}
**Supplementary Fig. 12**	
JK107	*h*$^-$
JP2247	*h*$^-$ *sgo2*::*hyg*^{r}
JP2251	*h*$^-$ *bub1*::*hyg*^{r}
JM2516	*h*$^-$ *taz1*::*kan*^{r}

Supplementary Table 2. Primers used to construct the strains

***sgo2* deletion** (PCR-based, other than Fig. 7)	
km1	5’-CTTTTTGAAACAGAGGAACAGATAG-3’
km2	5’-GGGGATCCGTCGACCTGCAGCGTACGAATAAATAGAAAATCCCGATGTACTTC-3’
km3	5’-GTTTAAACGAGCTCGAATTCATCGATTATGTAATTAAATACCTTTCGACATC-3’
km4	5’-AGACATATTATCGCCGTTATTAAAC-3’
***sgo2* deletion** (PCR-based, Fig. 7)	
HM2863	5’-CCACTGAAAGGATTAGTGAATGG-3’
HM2864	5’-ATCCGTCGACCTGCAGCGTATGTACTTCATTGACCACGTTCC-3’
HM2865	5’-AGATCCACTAGTGGCCTATGCTTTCGACATCGTTTAGTTGGTTC-3’
HM2866	5’-CTACTTGATGAATGTGCTAAGGTAC-3’
***bub1* deletion** (PCR-based)	
jk1021	5’-TTTGCTGCATCCAAGATGATGC-3’
jk1022	5’-GGGGATCCGTCGACCTGCAGCGTACGAGTGATACGCACATTGAAACATGG-3’
jk1023	5’-GTTTAAACGAGCTCGAATTCATCGATCCTAGAGGGAAGTTTCCATCC-3’
jk1024	5’-GTGCTGACCTCTGGCCTCG-3’
***hrk1* deletion** (PCR-based)	
tb75	5’-AGAAACTGTAACCCAGCCCAAGC-3’
tb76	5’-GGGGATCCGTCGACCTGCAGCGTACGAATCTTTTCCTACATTTGTAAATATTGAG-3’
tb77	5’-GTTTAAACGAGCTCGAATTCATCGATTTATGGTTTTTGCATACTAAAAAATTG-3’
tb78	5’-CTCAGCGCTATATTCAAGCTTGG-3’
***mph1* deletion** (PCR-based)	
jk1671	5’-CTTTAGGTGTTGTTTATCCTGAAAG-3’
jk1672	5’-GGGGATCCGTCGACCTGCAGCGTACGAAAACACAGTTACTAAAAAACGAGCA-3’
jk1673	5’-GTTTAAACGAGCTCGAATTCATCGATAGGCACTAAATTTTCCGATTATTCTT-3’
jk1674	5’-TTAGATTTAACTTAGATTTTTTCACAAG-3’
***taz1* deletion** (PCR-based)	
jk969	5’-TTGTTTCAGTTTGACGTTCTTGTTG-3’
jk970	5’-GGGGATCCGTCGACCTGCAGCGTACGATCTGTACTTTGCACGCTTATCATC-3’
jk967	5’-GTTTAAACGAGCTCGAATTCATCGATGTCTCCGGATAGAGTTTGTTCATG-3’
jk968	5’-TTGTGCTTCACTCATTTACGATTCC-3’
***dcr1* deletion** (PCR-based)	

jk639	5'-TAAAATAAAGCATAGGCGTTT-3'
jk640	5'-TTAATTAACCCGGGGATCCGAGGCGGAAGGTTTCATTCAATTG-3'
jk641	5'-GTTTAAACGAGCTCGAATTCGTTAAAAGTTTGACTTGAGACTTG-3'
jk642	5'-AGCAGCTTACGAATCGAAAGAG-3'
***clr4* deletion** (PCR-based)	
jk1467	5'-AGTAATATCCTAACATATGACATATAG-3'
jk1468	5'-GGGGATCCGTCGACCTGCAGCGTACGACGCAAAACTAATAACCTCTTGTTTG-3'
jk1469	5'-GTTTAAACGAGCTCGAATTCATCGATCCACTCCTGTTCACCTAATATAGC-3'
jk1470	5'-CTTCACATGTGCGCAGCAGATG-3'
***set2* deletion** (PCR-based)	
jk1675	5'-TTCAAAGATATTCGCTACATAAGGC-3'
jk1676	5'-GGGGATCCGTCGACCTGCAGCGTACGATGTATGATAATGGACAAAAAGAAAGAG-3'
jk1677	5'-GTTTAAACGAGCTCGAATTCATCGATTCGCCAGTATTTTTAACTGCGACA-3'
jk1678	5'-TCACCGTTTACAGATTTATTACTCG-3'
***sgo2* tagging** (PCR-based, other than Supplementary Fig. 6)	
km5	5'-CATCCCAATAATTCTCCGTTACG-3'
km6	5'-GGGGATCCGTCGACCTGCAGCGTACGACAAATTAAGGGTTTCGGAGTTTTC-3'
km3	5'-GTTTAAACGAGCTCGAATTCATCGATTATGTAATTAAATACCTTTCGACATC-3'
km4	5'-AGACATATTATCGCCGTTATTAAAC-3'
***sgo2* tagging** (PCR-based, Supplementary Fig. 6)	
HM3008	5'-AAGGAGCCGAAGGGAACGGAA-3'
HM3009	5'-CCGTGATGATGATGGTGGTGCAAATTAAGGGTTTCGGAGTTTTCA-3'
HM3010	5'-CGCCATCCAGTTTAAACGAGCCTTTCGACATCGTTTAGTTGG-3'
HM2866	5'-CTACTTGATGAATGTGCTAAGGTAC-3'
***gar1* tagging** (PCR-based)	
jk1514	5'-ATGGTTTGCCAGTCAACCAATG-3'
jk1515	5'-GGGGATCCGTCGACCTGCAGCGTACGAGAATCTGCCACGGAAACCACC-3'
jk1516	5'-GTTTAAACGAGCTCGAATTCATCGATTGGATAAGTATATAAAAGGTTTCG-3'
jk1517	5'-TTATTGTGTGGACTGATGACCG-3'
***nbl1* tagging** (PCR-based)	
tb30	5'-AACATCGTCAAGTACGACAGCGAATTC-3'
tb31	5'-GGGGATCCGTCGACCTGCAGCGTACGACACTTGCGACTTTGAACTCGATTT-3'
tb32	5'-GTTTAAACGAGCTCGAATTCATCGATAACCCTCATCTACTGCTTACTGTG-3'

tb33	5'-AATGGATGGAAATAGGCAAACTTGAAAAC-3'
***taz1* tagging** (Plasmid-based)	
jk969	5'-TTGTTTCAGTTTGACGTTCTTGTTG-3'
jk970	5'-GGGGATCCGTCGACCTGCAGCGTACGATCTGTACTTTGCACGCTTATCATC-3'
***ark1* tagging** (Plasmid-based)	
jk1173	5'-TATAAAAGATCTCCCTTCGTAAGATAGAAGAGCC-3'
jk1174	5'-TATAAAGCGGCCGCAGGAAGATTCAGAACTTTTGCGAG-3'
***pic1* tagging** (Plasmid-based)	
jk1204	5'-TATAAGGTACCACGCGTCACGTCGAAACCC-3'
jk1205	5'-TATAAAGCGGCCGCATAAAAAACCCATGTTTTTCTTATAG-3'

Supplementary Table 3. Primer sets used for the quantitative PCR analyses performed in this study

his1 (Figs. 1, 2, 3, 4, 5, 6, Supplementary Figs. 3, 7, 9)	
jk1335	5'-CGAAGACGTGCTTCAGCGA-3'
jk1336	5'-TGTCCACCTCGGAATCACTG-3'
cen(dg) (Figs. 1, 2, 3, 5, Supplementary Figs. 7, 9)	
st17	5'-AATTGTGGTGGTGTGGTAATAC-3'
st18	5'-GGGTTCATCGTTTCCATTCAG-3'
cen(dg) (Supplementary Fig. 6)	
	5'- TCCAAATGTCGCATGAACACTC-3'
	5'- CTTTTTTGGGAATACATTGGGTTT-3'
rDNA (Fig. 1)	
TB19	5'-GGGAACCAGGACTTTTACCTTGA-3'
TB20	5'-AACTTGCCTGCTTTGAACACTCTA-3'
ura4 (Fig. 6)	
st63	5'-GTATTTCCAATGTCTGCGAATTTG-3'
st64	5'-CATATTGACGTTGTCGAGGATTTC-3'
ade6 (Supplementary Fig. 6)	
	5'-TGATGGAGGACGTGAGCACATTGA-3'
	5'-TTGAATGCATCGCAGAGTTGCAGG-3'
subtel2R-0.2 kb (Supplementary Fig. 3)	
jk380	5'-TATTTCTTTATTCAACTTACCGCACTTC-3'
jk381	5'-CAGTAGTGCAGTGTATTATGATAATTAAAATGG-3'
subtel2R-2.8 kb (Supplementary Fig. 3)	
jk614	5'-GTCTCGTTGCTCGCTTCACA-3'
jk615	5'-GGAGGATGGGAAATTTTGAGGAT-3'
subtel2R-6.3 kb (Supplementary Fig. 3)	
jk618	5'-GCCTACCGCTTGCAGTTGTT-3'
jk619	5'-GGTTTGAGCATCTGTCAGAGGTAA-3'
subtel2R-9.0 kb (Figs. 2, 3, 3, 4, Supplementary Figs. 3, 4)	
jk620	5'-TTCTTAATCATTATCAAGTATTCATTGCAA-3'
jk621	5'-ACAGTAAACTATGATCGCTTTTGAAGAC-3'

subtel2R-14.7 kb (Supplementary Fig. 3)

jk1607	5'-GCTTGGTCAACATCATCTTGTCG-3'
jk1608	5'-GGGAGCAGCAAGACAAAGGTG-3'

subtel2R-20.0 kb (Figs. 2, 3, 4, Supplementary Figs. 3, 4, 7)

jk455	5'-AACGAGTTGTGCAATGTTAGTAAGGT-3'
jk456	5'-GACCGCTACGCAACCATAAAG-3'

subtel2R-37.9 kb (Figs. 2, 3, 4, Supplementary Fig. 4)

jk690	5'-TGAAACGGGTTCTTACTGCGT-3'
jk691	5'-GCTCCATCCATTGTCATTGGT-3'

subtel2R-51.8 kb (Figs. 1, 2, 3, 4, Supplementary Figs. 4, 7)

jk700	5'-TCCGCAAATTTTGTTAGCCAT-3'
jk701	5'-AGCTTAATTCGTGATGCAAGTTTTTA-3'

subtel2R-71.6 kb (Figs. 2, 3, 4, Supplementary Fig. 4)

km28	5'-CAAAAGCACAAGGGTTGACCA-3'
km29	5'-CCCGACTCCCTCAACATGAA-3'

subtel2R-89.8 kb (Figs. 2, 3, 4, Supplementary Figs. 4, 7)

st110	5'-TGCGGACATCATAACATGCTAAA-3'
st111	5'-TTCAATATACACCGAGGTCCGAAT-3'

subtel2R-98.9 kb (Figs. 2, 3, 3, 4, Supplementary Fig. 4)

km38	5'-TTCAGGATTAAGGTAACGCGGT-3'
km39	5'-TTGCAGTTGTCCGCTAGTGC-3'

subtel2R-110.8 kb (Figs. 2, 3, 4, Supplementary Fig. 4)

km40	5'-CCTATGCCTACGCATTCAGCTC-3'
km41	5'-CGATCGTCCACTACTTCACGTTT-3'

subtel2R-122.8 kb (Figs. 2, 3, 4, Supplementary Fig. 4)

st106	5'-AATTTTACCGGCTTCGCATCT-3'
st107	5'-TGCGTTTTCTCCGTGAATGA-3'

***ght3* (*subtel1L-94.7 kb*)** (Fig. 5, Supplementary Fig. 9)

yn120	5'-TTTATCTCAATGTCCGGATGGTT-3'
yn121	5'-CCAAGATGCCGCTAATGGAA-3'

***pfl9* (*subtel1R-70.1 kb*)** (Fig. 5, Supplementary Fig. 9)

st49	5'-GCTCTGGCGCCTTTGTTATTAG-3'
st50	5'-CGCTTCGCTTTTGTATCTGTGT-3'

***mel1* (*subtel1R-97.3 kb*)** (Fig. 5, Supplementary Fig. 9)

TB27	5'-TTAGTCGTAAGGATGTGCGTTGTC-3'
TB28	5'-ATCTCCCTGAAGCGCAAAAC-3'

***ght7* (*subtel2L-69.0 kb*)** (Fig. 5, Supplementary Fig. 9)

st51	5'-AATTGACAGCATCCGGAATTAAG-3'
st52	5'-ATTCCGCGGTACCGGTATG-3'

***aes1* (*subtel2L-100.6 kb*)** (Fig. 5, Supplementary Fig. 9)

st37	5'-AAGCTTTGCGCCTTTAATACATG-3'
st38	5'-CGACAGCTCCTGCACCACTAC-3'

***gal1* (*subtel2R-53.5 kb*)** (Fig. 5, Supplementary Fig. 9)

jk702	5'-GCGCCTTGTTTTGCTCTCC-3'
jk703	5'-ACGGGCGGTATGGATCAAT-3'

non-ori (Fig. 7, Supplementary Fig. 11)

HM1913	5'-TACGCGACGAACCTTGCATAT-3'
HM1914	5'-TTATCAGACCATGGAGCCCATT-3'

ars2004 (Fig. 7, Supplementary Fig. 11)

HM1911	5'-CGGATCCGTAATCCCAACAA-3'
HM1912	5'-TTTGCTTACATTTTCGGGAACTTA-3'

AT2080 (Fig. 7)

HM1093	5'-CGAACAACAGGCTTGGTTAGAA-3'
HM1094	5'-GAAGTACGGACTTGTTTCGATTCC-3'

AT2088 (Fig. 7, Supplementary Fig. 11)

HM2555	5'-TCCTCACTTCCTTAAAAACAGATTAAGAAATA-3'
HM2556	5'-CGCAAAATAACATCGTAGTGGAAC-3'

tel-0.2 (Fig. 7, Supplementary Fig. 6)

HM1627	5'-TATTTCTTTATTCAACTTACCGCACTTC-3'
HM1628	5'-CAGTAGTGCAGTGTATTATGATAATTAAAATGG-3'

tel-11.8 (Fig. 7)

HM1635	5'-GCTCTCGACAAAGCCGTTCT-3'
HM1636	5'-CAGCATTAACCAACAGTGGTCTTC-3'

tel-21.2 (Fig. 7, Supplementary Fig. 6, 11)

HM1132	5'-CAGAAGAGACTACAGAGGCGGTTT-3'
HM1133	5'-GGATGCCTTATCTGCGACCA-3'

tel-30.1 (Fig. 7)

HM3096	5'-AGCAACATGGCAGCACCAT-3'
HM1133	5'-GATAATCGTAAAGCTGGGAATACCA-3'

tel-41.6 (Fig. 7, Supplementary Fig. 6)

HM1639	5'-CGCGCACTTTTCGGACATA-3'
HM1640	5'-TGCAAGTCGCCGAAACTACC-3'

tel-45.7 (Fig. 7)

HM1641	5'-TTGAATCCCTCATCCAAAGGA-3'
HM1642	5'-TTGGTGTCAGCCCATTGAACT-3'

tel-55.0 (Fig. 7)

HM2103	5'-AAACTGTGACTTTCTGCATCGAAT-3'
HM2104	5'-AATCCGGTAATCATAGCTTATAATAAAGC-3'

tel-60.0 (Fig. 7)

HM2055	5'-TGCTTCTGTCCATCTAGCTCTTGT-3'
HM2056	5'-CGTTGTTGAGATCAGCTACAGTGA-3'

tel-72.3 (Fig. 7)

HM2090	5'-AATTGAGGCATGTAGTGACTTCCA-3'
HM2091	5'-CCAGATGGTATCAACAACAAATAAATAGA-3'

tel-84.5 (Fig. 7)

HM2086	5'-AGACAATTTCAACGGCGAAGA-3'
HM2087	5'-AGTCCTTGTCCGGCAGAATCT-3'

tel-94.1 (Fig. 7)

HM2196	5'-GATCTTTGGAGGTGCCTATGCTA-3'
HM2197	5'-AAAGGCGAAGAAAACTTGTTGAGT-3'

tel-100.8 (Fig. 7)

HM2198	5'-AGAATGAGAGGAGAAACGAAGACTTTA-3'
HM2199	5'-CATCGTCCTTCGTAATTCTTGTCTTT-3'

Supplementary References

1. Sugawara, N.F. DNA sequences at the telomeres of the fission yeast *S. pombe. Ph.D. Thesis, Harvard University* (1988).

2. Nakamura, T.M., Cooper, J.P. & Cech, T.R. Two modes of survival of fission yeast without telomerase. *Science* **282**, 493-6 (1998).

3. Hayashi, M. et al. Genome-wide localization of pre-RC sites and identification of replication origins in fission yeast. *EMBO J.* **26**, 1327-39 (2007).

4. Tazumi, A. et al. Telomere-binding protein Taz1 controls global replication timing through its localization near late replication origins in fission yeast. *Genes Dev.* **26**, 2050-62 (2012).
